# Advancing age increases the size and severity of spontaneous atheromas in mouse models of atherosclerosis

**DOI:** 10.1007/s11357-023-00776-8

**Published:** 2023-04-22

**Authors:** Venkateswara R. Gogulamudi, Jessica R. Durrant, Adelola O. Adeyemo, Huynh Mi Ho, Ashley E. Walker, Lisa A. Lesniewski

**Affiliations:** 1 Internal Medicine-Geriatrics, University of Utah, Salt Lake City, UT USA; 2 Geriatrics Research Education and Clinical Center, Veteran’s Affairs Medical Center-Salt Lake City, Salt Lake City, UT USA; 3 Dallas Tissue Research, Dallas, TX USA; 4 Department of Human Physiology, University of Oregon, Eugene, OR USA; 5 Nutrition and Integrative Physiology, University of Utah, Salt Lake City, UT USA; 6 Nora Eccles Harrison Cardiovascular Research and Training Institute, University of Utah, Salt Lake City, UT USA

**Keywords:** Aging, Atherosclerosis, ApoE, Pcsk9, Animal models of human disease, Aging, Pathophysiology, Vascular biology, Atherosclerosis, Animal models of human disease, Aging, Pathophysiology, Vascular biology, Atherosclerosis

## Abstract

Using multiple mouse models, we explored the impact of aging on the size and severity of atherosclerotic lesions. In young, middle-aged and old apolipoprotein E knockout mice (ApoE^−/−^) fed an atherogenic diet (AD) for 3–8 weeks, plaque/atheroma formation in the descending aorta and aortic root, and atheroma development in the carotid in response to partial carotid ligation (PCL) were assessed. Total and LDL cholesterol, and triglycerides were higher in old compared to both other age groups, regardless of AD duration. Aortic plaque burden increased with AD duration in all ages. The size and plaque morphology grade of aortic root atheromas was higher with age; however, there was no effect of age on the size or severity of carotid atheromas after PCL. We additionally induced hyperlipidemia in young and old C57BL/6 mice by adeno-associated virus mediated upregulation of LDL receptor regulator, Pcsk9, and 5 weeks of AD. Despite lower cholesterol in old compared to young *Pcsk9* mice, there was a greater size and severity of aortic root atheromas in old mice. However, like the ApoE^−/−^ mice, there was no effect of age on size or severity of PCL-induced carotid artery atheromas in *Pcsk9* mice. Together, these results suggest that aging increases the size and severity of spontaneous aortic atheromas.

**Supplementary Information:**

The online version contains supplementary material available at 10.1007/s11357-023-00776-8.

## Introduction

Cardiovascular disease (CVD) is the leading cause of mortality in the US, accounting for ~ 25% of deaths [1, 2] and over $350 billion in health care costs [3] each year. Advanced age is the primary risk factor for CVD [4] with the morbidity and mortality associated with CVD increasing as a consequence of aging [2]. Atherosclerotic CVD leads to myocardial infarction, stroke, and peripheral vascular diseases, which are increasingly prevalent in older adults [2, 5–7] and contribute significantly to morbidity and mortality associated with CVD. Understanding mechanisms for the age-associated increase in atherosclerotic disease may better direct prevention and therapeutics in this at-risk population. Characterizing the impact of aging in animal models of atherosclerotic disease is critical to this effort.

In non-atheroprone mouse models, vascular aging is characterized by endothelial dysfunction and large artery stiffening [8–11], both of which are independent risk factors for atherosclerotic CVD [12, 13]. Although this relation between age-associated vascular dysfunction and CVD is well recognized, it is unclear if aging per se increases susceptibility to and severity of atherosclerotic disease. An atherogenic diet (AD) induces higher circulating cholesterol and a more pro-inflammatory arterial phenotype, evidenced by higher vascular cell adhesion molecule-1 (VCAM-1) expression, in old compared to young C57BL/6 mice. However, this increase in cholesterol and arterial inflammation in response to an AD was not associated with an increase in fatty streak area in old control mice [14]. Our laboratory has reported similar findings, in which we assessed neointima formation in response to surgically-induced oscillatory shear in C57BL/6 mice fed a high fat diet and observed a similar proliferative response between young and old mice [15]. Although these findings suggest that aging may not impact atherosclerotic disease susceptibility, others have found that aging (from 12–38 weeks of age) increases size and complexity of atherosclerotic lesions in apolipoprotein E knockout (ApoE^−/−^) mice, suggesting that atherogenic responses to pathological hyperlipidemia may be exacerbated by aging [16]. However, this increase in lesion development with aging in the ApoE^−/−^ mouse may be the consequence of the prolonged hyperlipidemic stimulus rather than an effect of aging per se [16]. Thus, longitudinal studies of the effect of aging in the ApoE^−/−^ model are confounded by the length of time mice are exposed to the genetically induced atherogenic, hyperlipidemic stimulus, making a true evaluation of the impact of aging difficult.

In this study, we aimed to explore the impact of aging on the size and severity of atherosclerotic lesions in multiple atheroprone mouse models: ApoE^−/−^ and adeno-associated virus (AAV)-proprotein convertase subtilisin/kexin type 9 (*Pcsk9*) treated C57BL/6 mice. To do so, we examined plaque burden in the descending aorta, and atheroma size and plaque morphology grade in the aortic root, as well as in the left carotid artery five weeks after induction of oscillatory shear stress via partial carotid ligation (PCL) in young, middle-aged, and old ApoE^−/−^ mice. We also treated young and old C57BL/6 mice with AAV containing a modified *Pcsk9* gene to acutely induce hyperlipidemia and assessed the same outcomes. We hypothesized that aging would exacerbate plaque burden in the descending aorta as well as increase the size and worsen the plaque morphology grade of atheroma in the aortic root and left carotid arteries after PCL.

## Results

### Circulating lipids in young, middle-aged and old ApoE^−/−^ mice

**Fig. 1:**
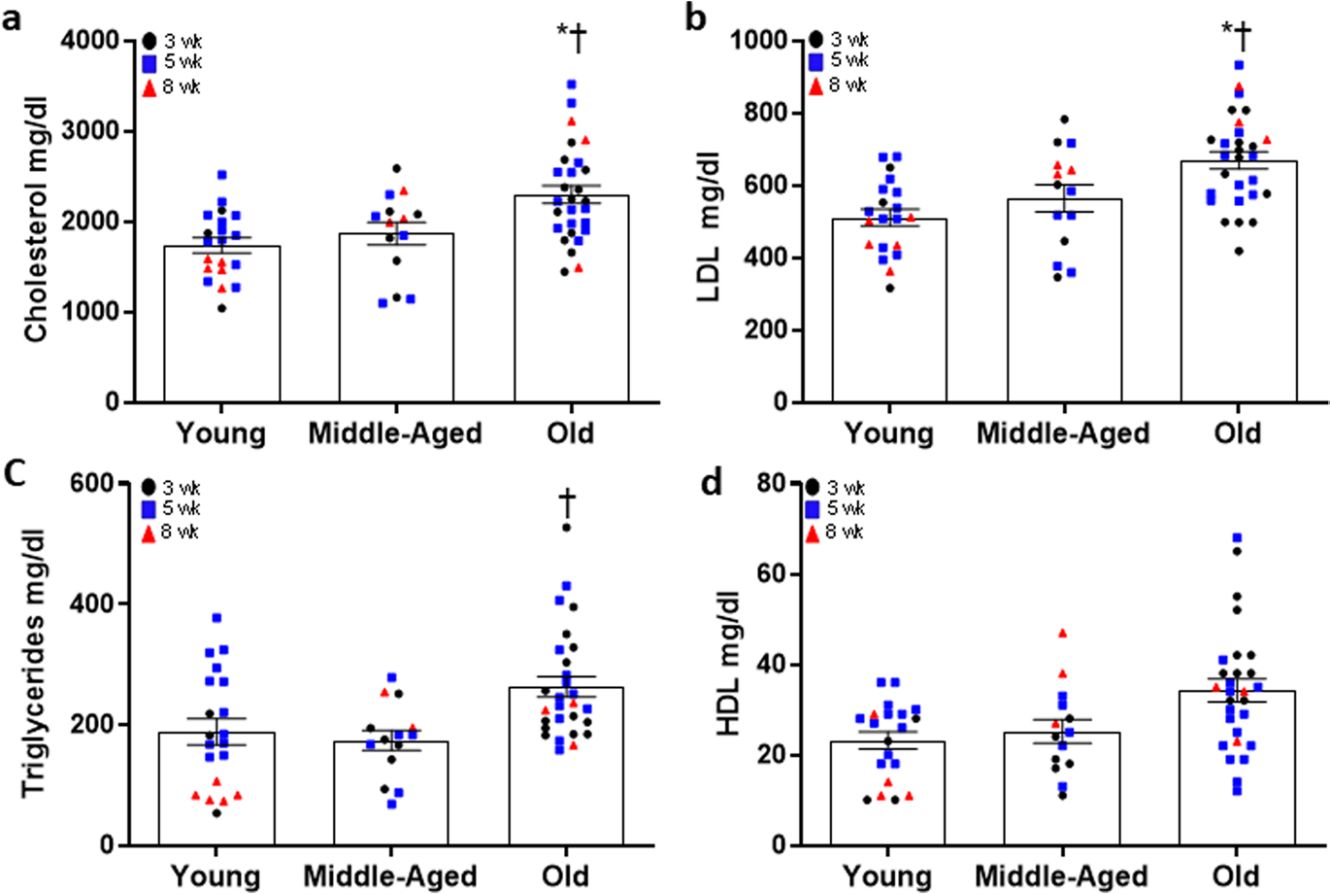
Circulating lipids in young (6.6 ± 0.1 mo, *N* = 20), middle-aged (10.1 ± 0.2 mo, *N* = 14) or old (16.9 ± 0.1 mo, *N* = 28) apolipoprotein E knockout (ApoE^−/−^) mice fed an atherogenic diet (AD) for 3, 5, or 8 weeks. Total cholesterol (a), LDL cholesterol (b), triglycerides (c), and HDL cholesterol (d) were assessed by an Architect ci8200 biochemical analyzer. Data presented are combined over weeks and raw values for young, middle-aged, and old mice after 3 (black circles), 5 (blue squares), and 8 (red triangles) wk AD. Summary data are mean ± SEM,* denotes *P* < 0.05 compared to young when data is combined over weeks on AD, † denotes *P* < 0.05 compared to middle-aged when data is combined over weeks on AD. To assess the difference between age groups and when these data were combined over weeks on AD, a one-way ANOVA with least square difference (LSD) post hoc testing was performed

We assessed circulating lipids and atherosclerotic development in young (6.6 ± 0.1 mo, M/F:17/8), middle-aged (10.1 ± 0.2 mo, M/F: 13/3) and old (16.9 ± 0.1 mo, M/F: 18/14) ApoE^−/−^ mice fed an AD for 3, 5, or 8 weeks. Although there was no effect of diet duration on total (*P* = 0.352), LDL (*P* = 0.574), and HDL (*P* = 0.687) cholesterol, or triglycerides (*P* = 0.244), there was a main effect of age for triglycerides (*P* = 0.038), total (*P* < 0.001) and LDL (*P* = 0.002), but not HDL cholesterol (*P* = 0.127) (Fig. 1a-d). When combining data over weeks on AD, total (Fig. 1a, both *P* ≤ 0.015) and LDL (Fig. 1b, both *P* ≤ 0.049) cholesterol were higher in old compared to middle-aged and young mice. There were no differences between young and middle-aged mice for total (*P* = 0.582) or LDL (*P* = 0.520) cholesterol. Triglycerides were higher in old compared to middle-aged (Fig. 1c, *P* = 0.008), but not young (*P* = 0.064) mice on AD for 3–8 weeks. There was no difference in triglycerides between young and middle-aged mice (*P* = 0.270). HDL cholesterol did not differ among age groups (Fig. 1d, *P* = 0.127). Thus, an age-associated increase in circulating total and LDL cholesterol may contribute to increases in plaque burden, size, and severity. We observed similar trends in different diet durations in each age group (Supplementary Fig. 1).

### Plaque burden in the descending aorta and size and severity of aortic root atheromas in ApoE^−/−^ mice

**Fig. 2:**
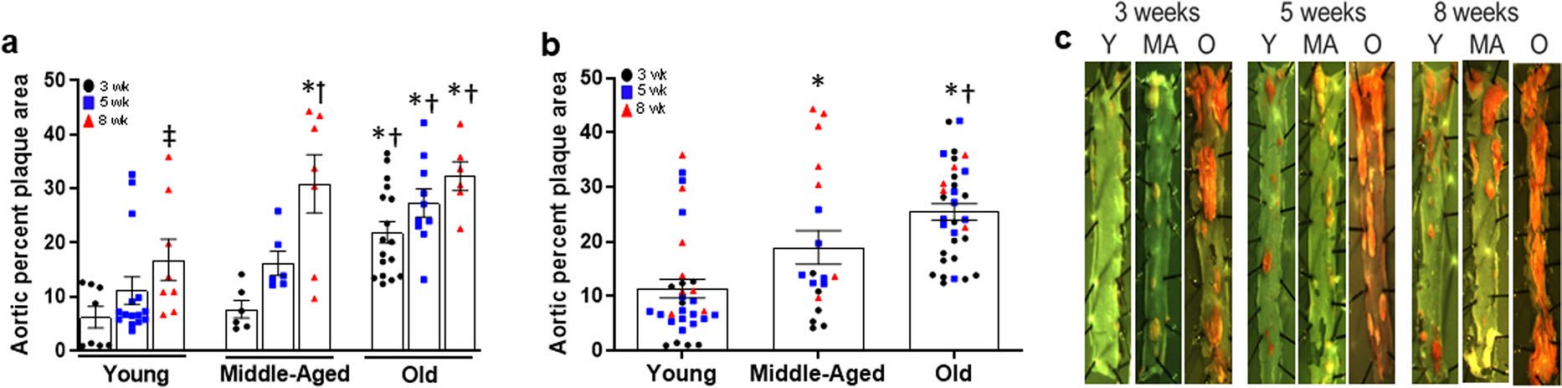
Aortic plaque burden in the descending aorta of young (Y: *N* = 31), middle-aged (MA: *N* = 19) and old (O: *N* = 33) apolipoprotein E knockout (ApoE-/-) mice fed an atherogenic diet (AD) for 3, 5, or 8 weeks (wk). Aortic plaque burden was assessed by quantifying percent plaque area in digital images of Sudan IV stained aortas using imageJ software. (a) Aortic percent plaque area in young, middle-aged and old ApoE^−/−^ mice after 3, 5 or 8 weeks on AD. * denotes *p* < 0.05 compared to young within AD group, † denotes *p* < 0.05 compared to middle-aged within AD group, ‡ denotes difference from 3 wks within age group, § denotes difference from 5 wks within age group. (b) Aortic percent plaque area in young, middle-aged, and old mice when data is combined over weeks on AD. Individual data presented are raw values for young, middle-aged, and old mice after 3 (black circles), 5 (grey squares), and 8 (red triangles) wk AD. Summary data are mean ± SEM, * denotes *p* < 0.05 compared to young when data is combined over weeks on AD, † denotes *p* < 0.05 compared to middle-aged when data is combined over weeks on AD. (c) Representative images of Sudan IV stained descending aortas. To assess the difference between age groups and diet duration for measures, a two-way ANOVA was performed and LSD post hoc testing. When these data were combined over weeks on AD, a one-way ANOVA with LSD post hoc testing was performed

Aortic plaque burden was assessed in the longitudinal descending aorta by quantifying lipid deposition after Sudan IV staining. There was a main effect for age (*P* < 0.001) and diet duration (*P* = 0.001), but not a significant interaction (*P* = 0.275) for aortic plaque burden. In young and old ApoE^−/−^ mice, there was an increased plaque burden in the aorta after 8 weeks on AD compared to 3 (*P* = 0.018), but not 5 (*P* = 0.201), weeks on AD (Fig. 2a). In middle-aged mice, plaque burden was higher after 8 weeks on AD compared to both 3 (*P* < 0.001) and 5 (*P* = 0.004) weeks on AD (Fig. 2a). Plaque burden were higher in middle-aged mice after 8 weeks on AD compared to diet duration-matched young mice (Fig. 2a, *P* < 0.001). Plaque burden was also higher in old compared to young mice at 3 (*P* < 0.001), 5 (*P* < 0.001) and 8 (*P* < 0.001) weeks on AD (Fig. 2a) and greater than in middle-aged mice at 3 weeks on AD (*P* = 0.001) (Fig. 2a). When plaque burden data were collapsed over weeks on AD, both middle-aged (*P* = 0.004) and old mice (*P* < 0.001) demonstrated higher plaque burden than young mice (Fig. 2b), and old mice had a greater plaque burden when compared to middle-aged mice (Fig. 2b, *P* = 0.012). Representative images of plaques in the descending aorta are presented in Fig. 2c.

**Fig. 3:**
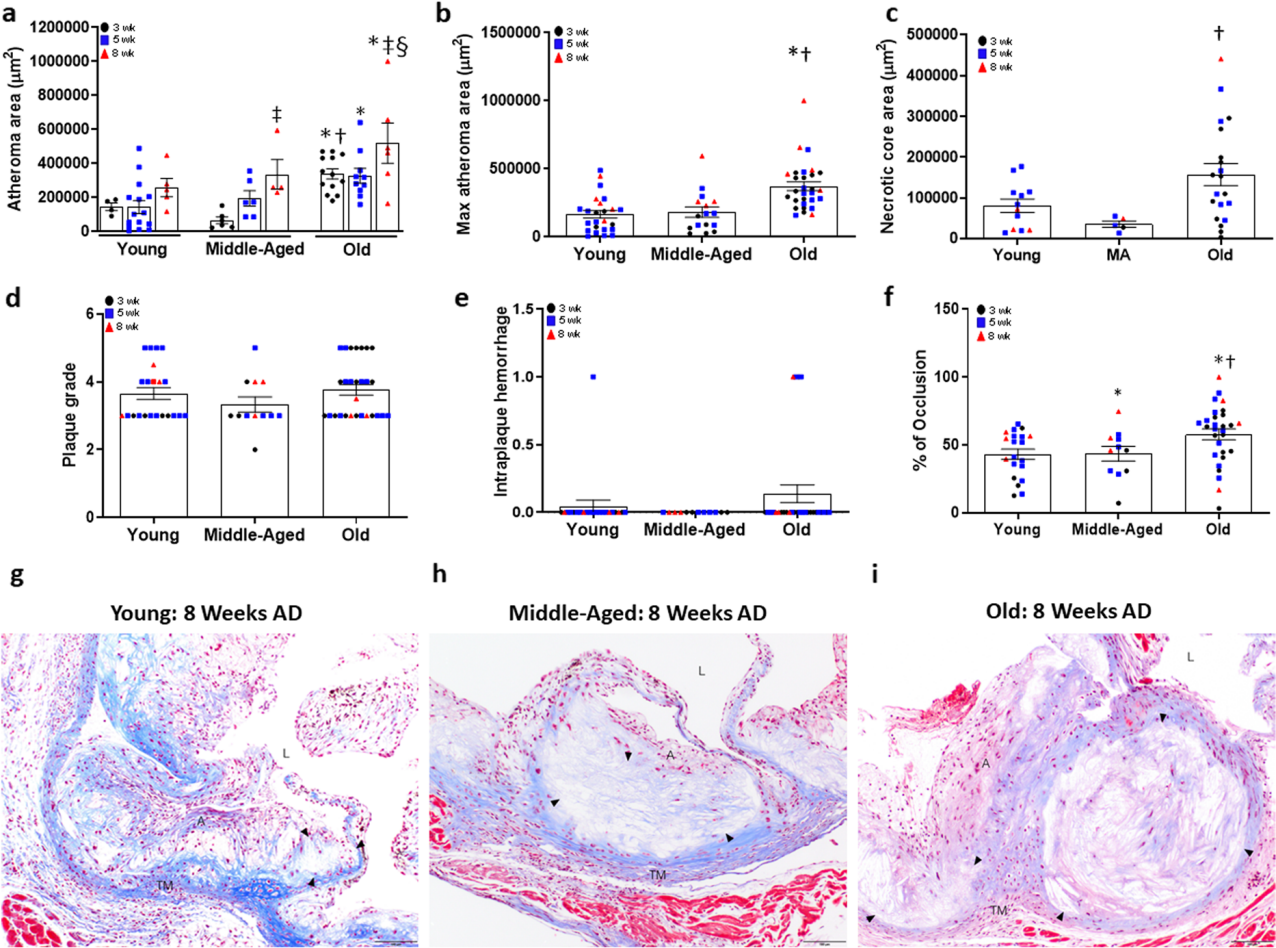
Size and severity of atheromas in the aortic root of young (Y: *N* = 12–24), middle-aged (MA: *N* = 5–16) and old (O: *N* = 29) apolipoprotein E knockout (ApoE^−/−^) mice fed an atherogenic diet (AD) for 3, 5, or 8 weeks. (a) Atheroma area was measured in Maisson’s trichrome stained histological sections of aortic roots from young, middle-aged and old ApoE^−/−^ mice after 3, 5 or 8 weeks on AD. * denotes *p* < 0.05 compared to young within AD group, † denotes *p* < 0.05 compared to middle-aged within AD group, ‡ denotes difference from 3 wks within age group, § denotes difference from 5 wks within age group. (b) Aortic root atheroma area in young, middle-aged, and old mice when data is combined over weeks on AD. (c) Area of necrotic cores, when present, in aortic root atheromas from young, middle-aged, and old mice when data is combined over weeks on AD. (d) Plaque morphology grade and severity score. (e) Intraplaque hemorrhage in aortic root atheromas from young, middle-aged and old mice when data is combined over weeks on AD. (f) Lumenal occlusion expressed as the percent reduction by atheroma within the aortic root intimal area, measured with Masson’s trichrome-stained slides. Individual data presented are raw values for young, middle-aged, and old mice after 3 (black circles), 5 (grey squares), and 8 (red triangles) wk AD. Summary data are presented as mean ± SEM. * Denotes *P* < 0.05 compared to young when data is combined over weeks on AD, † denotes  *P*< 0.05 compared to middle-aged when data is combined over weeks on AD. Representative images of Masson’s trichrome stained images of aortic roots from (g) young, (h) middle-aged, and (i) old ApoE^−/−^ mice after 8wk AD. Lumen (L), atheroma (A), necrotic core (black arrowheads), and tunica media (TM) are indicated. Scale bar indicates 100 μm. To assess differences in plaque size and severity scores for plaque characteristics, nonparametric Mann–Whitney Wilcoxon signed rank tests were used

The size of the aortic root atheroma and necrotic core were assessed in Masson’s trichrome stained sections of paraffin embedded aortic roots. A main effect existed for age (*P* < 0.001) and diet duration (*P* = 0.001), but not a significant interaction (*P* = 0.733) for aortic root atheroma area. There was no effect of AD duration on aortic root atheroma area in young ApoE^−/−^ mice (Fig. 3a, *P* = 0.220). In middle-aged mice, aortic root atheroma area was larger after 8 weeks compared to 3 weeks on AD (Fig. 3a, *P* = 0.008). In old mice, atheroma area was higher after 8 weeks compared to both 3 and 5 weeks AD (Fig. 3a, both *P* ≤ 0.034). Atheroma area was larger in old compared to young mice at 3, 5 and 8 weeks AD (Fig. 3a, all *P* ≤ 0.021) and was larger in old compared to middle-aged mice after 3 weeks AD (Fig. 3a, *P* = 0.001). When combined over AD duration, aortic root atheroma area was larger in old mice compared to young and middle-aged mice (Fig. 3b, both *P* < 0.001).

To evaluate plaque stability, we assessed the presence and size of the atheroma’s necrotic core and overlying fibrous caps. In aortic root atheromas combined over weeks on AD, 13% (3 of 23) of young, 31% (5 of 16) of middle-aged, and 30% (9 of 30) of old ApoE^−/−^ mice contained a necrotic core and the size of this necrotic core tended to be larger in old compared with middle-aged mice (Fig. 3c, *P* = 0.041). Among aortic root atheromas from young mice, no fibrous caps were observed in atheromas after 3 or 5 weeks AD, but were present in 60% (3 of 5) of atheromas after 8 weeks AD. In middle-aged mice, 17% (1 of 6) of the atheromas after 3 weeks AD, 33% (2 of 6) atheromas after 5 weeks, and 40% (2 of 5) atheromas after 8 weeks of AD exhibited a fibrous cap overlying the necrotic core (Table 1). In old mice, 36% (5 of 14) of atheromas after 3 weeks, 23% (3 of 13) after 5 weeks, and 25% (1 of 4) of atheromas after 8 weeks of AD demonstrated a cap. When data for fibrous cap thickness was combined over weeks on diet, there was no effect of age on either minimal (young: 20.6 ± 2.5 µm, middle-age: 17.5 ± 7.5 µm, old: 22.7 ± 3.5 µm, all *P* = 0.700) or maximal cap thickness (young: 40.5 ± 8.2 µm, middle-age: 41.8 ± 9.7 µm, old: 46.3 ± 3.4 µm, all *P* = 0.729) (Table 1).

**Table 1:** Atherosclerotic development and plaque severity analysis of atheromas in the aortic root and 5 weeks after partial carotid ligation (PCL) in the left carotid artery of young (Y), middle-aged (MA) and old (O) apolipoprotein E knockout (ApoE-/-) mice fed an atherogenic diet (AD), and young (Y) or old (O) C57BL/6 mice treated with a control or proprotein convertase subtilisin/kexin type 9 (*Pcsk9*) mutant-containing adenoassociated virus (AAV) and fed AD

ApoE^−/−^ Mice											
Aortic Root											
		Group			*P* values						
		Y	MA	O	main effect	Y v MA	MA v O			Y v O	
	N	20	16	28							
Morphology Grade	Median	3	3	3	*0.041*	0.940	0.619			*0.036*	
	Min–Max	3–5	2–5	3–5							
Necrosis	Median	0	0	0	0.956	---	---			---	
	Min–Max	0–1	0–2	0–1							
Mineralization	Median	0	0	0	0.126	---	---			---	
	Min–Max	0–1	0–2	0–2							
Hemorrhage	Median	0	0	0	*0.026*	1.00	*0.049*			0.114	
	Min–Max	0–1	0–0	0–2							
Thrombosis	Median	0	0	0	1.000	---	---			---	
	Min–Max	0–0	0–0	0–0							
Recanalization	Median	0	0	0	1.000	---	---			---	
	Min–Max	0–0	0–0	0–0							
Inflammation	Median	1	1	1	0.604	---	---			---	
	Min–Max	0–3	0–2	0–3							
Carotid Artery											
		Group			*P* values						
		Y	MA	O	main effect	Y v MA	MA v O			Y v O	
	N	8	4	6							
Morphology Grade	Median	4.5	2.5	3.75	0.125	---	---			---	
	Min–Max	3–5.5	2–4	3–5.5							
Necrosis	Median	0	0	3	0.086	---	---			---	
	Min–Max	0–5	0–0	0–5							
Mineralization	Median	0	0	0	0.700	---	---			---	
	Min–Max	0–1	0–0	0–2							
Hemorrhage	Median	0	0	0.5	0.246	---	---			---	
	Min–Max	0–1	0–0	0–2							
Thrombosis	Median	0	0	0	0.359	---	---			---	
	Min–Max	0–5	0–0	0–4							
Recanalization	Median	0	0	0	0.445	---	---			---	
	Min–Max	0–2	0–0	0–4							
Inflammation	Median	1	0.5	1	0.203	---	---			---	
	Min–Max	1–2	0–2	1–1							
Pcsk9-AAV Treated C57BL/6J Mice											
Aortic Root											
		Group				P values					
		CON		Pcsk9-AAV		Main effect	Con v Pcsk9-AAV		Y v O		
							Y	O	Con		Pcsk9
		Y	O	Y	O						
	N	9	8	13	8						
Morphology Grade	Median	0	0	0	1.5	*0.001*	0.225	*0.005*	1.000		0.556
	Min–Max	0–0	0–0	0–2	0–3						
Necrosis	Median	0	0	0	0	1.000	---	---	---		---
	Min–Max	0–0	0–0	0–0	0–0						
Mineralization	Median	0	0	0	0	0.290	---	---	---		---
	Min–Max	0–0	0–0	0–0	0–1						
Hemorrhage	Median	0	0	0	0	1.000	---	---	---		---
	Min–Max	0–0	0–0	0–0	0–0						
Thrombosis	Median	0	0	0	0	1.000	---	---	---		---
	Min–Max	0–0	0–0	0–0	0–0						
Recanalization	Median	0	0	0	0	1.000	---	---	---		---
	Min–Max	0–0	0–0	0–0	0–0						
Inflammation	Median	0	0	0	0	*0.008*	1.000	*0.034*	1.000		*0.014*
	Min–Max	0–0	0–0	0–0	0–1						
Carotid Artery											
		Group				*P* values					
		CON		Pcsk9-AAV		Main effect	Con v Pcsk9-AAV		Y v O		
							Y	O	Con		Pcsk9
		Y	O	Y	O						
	N	8	9	14	5						
Morphology Grade	Median	0	0	2.5	2	*0.000*	*0.010*	*0.028*	1.000		1.000
	Min–Max	0–0	0–0	0–4.5	2–2						
Necrosis	Median	0	0	0	0	0.666	---	---	---		---
	Min–Max	0–0	0–0	0–2	0–0						---
Mineralization	Median	0	0	0	0	1.000	---	---	---		---
	Min–Max	0–0	0–0	0–0	0–0						
Hemorrhage	Median	0	0	0	0	*0.015*	1.000	*0.021*	1.000		0.231
	Min–Max	0–0	0–0	0–1	0–2						
Thrombosis	Median	0	0	0	0	0.666	---	---	---		---
	Min–Max	0–0	0–0	0–2	0–0						
Recanalization	Median	0	0	0	0	0.172	---	---	---		---
	Min–Max	0–0	0–0	0–3	0–0						
Inflammation	Median	0	0	0.5	0	*0.046*	0.074	1.000	1.000		1.000
	Min–Max	0–0	0–1	0–2	0–1						

Aortic roots and lesions from left carotid arteries were assessed for plaque morphology grade according to modified AHA classification and assigned a severity score (0 = absent, 1 = minimal, 2 = mild, 3 = moderate, 4 = marked, 5 = severe) for relevant plaque characteristics including intimal necrosis, mineralization, intraplaque hemorrhage, recanalization, and inflammation. Data was collapsed over weeks on AD. Significant *P* values in italics

Plaque severity scoring was performed by a veterinary pathologist blinded to sample groupings according to modified AHA classifications and differences between groups were determined by nonparametric, Mann–Whitney Wilcoxon tests. Atheromas were assigned a plaque morphology grade as well as severity scores (0–5: absent-severe) for relevant plaque characteristics including intimal necrosis, mineralization, intraplaque hemorrhage, recanalization, and inflammation. The development of atheromas at the aortic root extended from the valve annulus into the ascending aorta. Atheroma morphology ranged from intimal xanthomas (Grade 2: aggregates of foam cells), to areas of pathologic intimal thickening (Grade 3: proteoglycan matrix, foam cells, and lipid/cholesterol clefts), to fibrous cap atheromas (Grades 4–5: pathologic intimal thickening in addition to necrotic cores capped by lamellar collagen bands). Layered lesions were often present, in which aggregates of foam cells were visible lumenal to intimal lesions or capped atheromas. Although small areas of mineralization were seen, lesions characterized as calcified nodules (Grade 6) and fibrocalcific plaques (Grade 7) were not specifically observed.

To determine if there was a significant interaction between age and weeks on diet for aortic root atheroma area, we first performed the analysis with both age and weeks on diet as independent variables. Although we found a significant main effect for age (*P* = 0.012), post hoc analyses did not find any differences between weeks on diet for any age group (asymptotic pairwise comparisons with Bonferroni correction all ≥ *P* = 0.086). As a result, all additional analyses were performed with weeks on diet data combined within each age group. When plaque morphology grades were combined over weeks on AD for the age groups, plaque morphology grade of aortic root atheromas was not different in old compared to young ApoE^−/−^ mice (Fig. 3d). No differences in plaque morphology grade were found between middle-aged and young (*P* = 0.940) or old (*P* = 0.619) mice. When collapsed over weeks on AD, there was a main effect of age (*P* = 0.026) for intraplaque hemorrhage, with the severity score not different in old compared to middle-aged (*P* = 0.244) or young (*P* = 0.429) mice (Fig. 3e and Table 1). Furthermore, when combined over the weeks on AD, there was an effect of age for percent lumenal occlusion (*P* = 0.008), with occlusion higher in middle-aged (*P* = 0.024) and old (*P* = 0.005) compared to young mice (Fig. 3f). No other differences in plaque characteristics were found (Table 1). Representative images of aortic root atheromas are presented in Fig. 3 g-i.

**Fig. 4:**
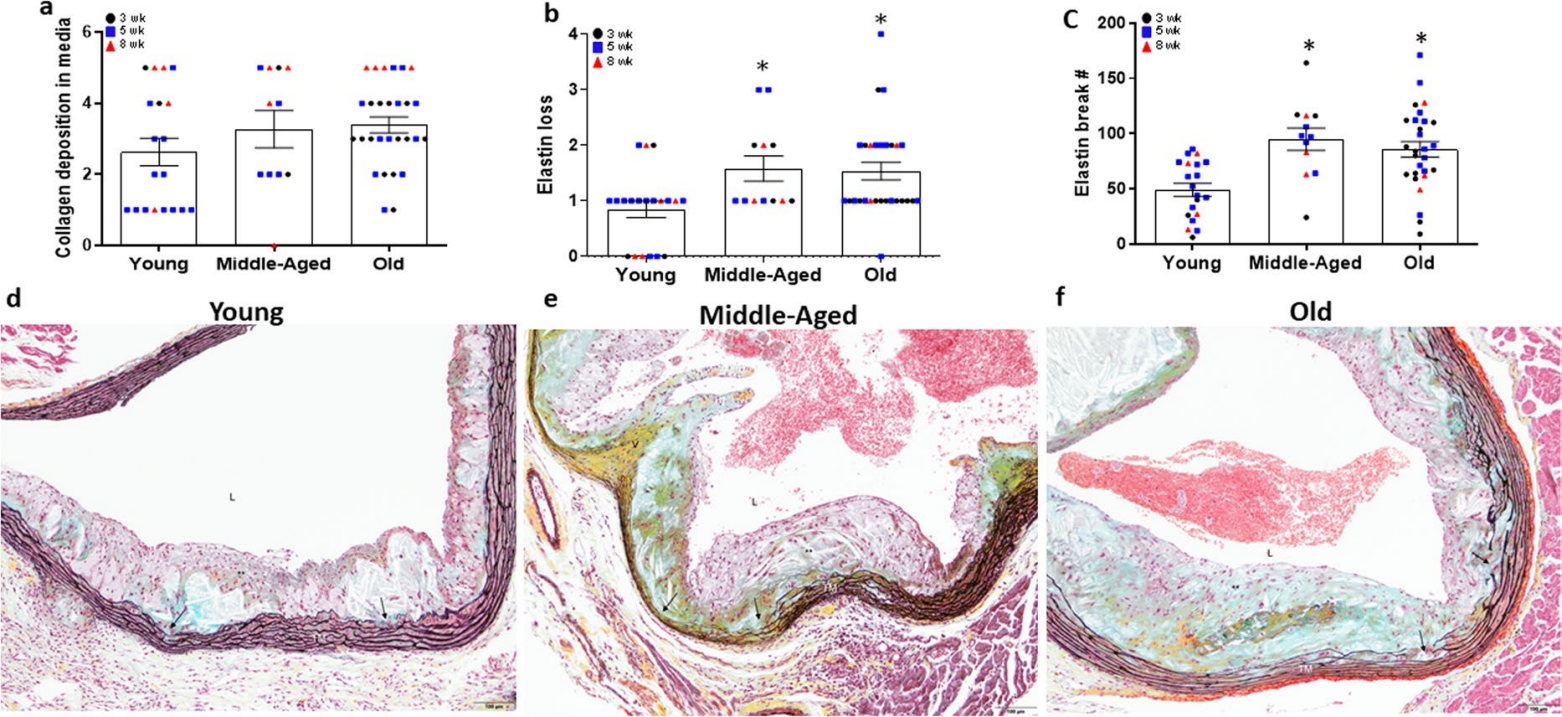
Measures of VSMC replacement by collagen or proteoglycan matrix and elastin loss in the aortic root of young (Y: *N* = 19–20), middle-aged (MA: *N* = 11–12) and old (O: *N* = 28) apolipoprotein E knockout (ApoE^−/−^) mice fed an atherogenic diet (AD) for 3, 5, or 8 weeks. (a) Area of tunica media VSMC replaced by proteoglycan (aqua color) or collagen (yellow color) matrix was assessed in Movat’s pentachrome-stained histological sections of aortic roots from young, middle-aged and old ApoE^−/−^ mice after 3, 5 or 8 weeks on AD. * denotes *p* < 0.05 compared to young within AD group. For samples with multiple aortic root atheroma samples available, maximal area was used in the analyses. (b) Elastin loss score (area of complete loss of elastin), as well as (c) elastin break counts were assessed using Movat’s pentachrome-stained sections. Individual data presented are raw values for young, middle-aged, and old mice after 3 (black circles), 5 (grey squares), and 8 (red triangles) wk AD. Representative images of Movat’s pentachrome-stained images of aortic roots from (d) young, (e) middle-aged, and (f) old ApoE^−/−^ mice after 8wk AD. Lumen (L), tunica media (TM), valve base (V), VSMC (stained red–orange), proteoglycan (stained aqua blue), collagen (stained yellow) matrix, elastin (laminae stained black), and areas of elastin loss and collagen/proteoglycan deposition (black arrows) are indicated. Scale bar indicates 100 μm. To assess differences in root morphology for plaque characteristics, nonparametric or Mann–Whitney Wilcoxon signed rank tests were used. Summary data is presented as mean±SEM

We next examined alterations in mural morphology in Movat’s stained sections of aortic root atheromas in mice after 3–8 weeks of AD. We assessed collagen deposition in the media of aortic roots with plaques and found no main effect for age (*P* = 0.132) (Fig. 4a). In contrast, elastin loss was greater in old (Fig. 4b, *P* = 0.004) and middle-aged mice (*P* = 0.015) compared to young mice. Similarly, there was a main effect for age in elastin breaks (*P* = 0.001), with a higher number of breaks in middle-aged (*P* = 0.001) and old (*P* = 0.001) compared with young group mice (Fig. 4c). Representative images of aortic root morphology including VSMCs, collagen, proteoglycan and elastin in young, middle-aged and older mice are presented in Fig. 4d-f.

### Partial carotid ligation’s effect of carotid artery lesions in ApoE^−/−^ mice

PCL was combined with 5 weeks of AD to impose an acute increase in oscillatory shear stimulus and induce atherosclerotic plaques in otherwise, non-atheroprone carotid arteries of young, middle-aged and old ApoE^−/−^ mice. As in the aortic root, carotid lesion morphology ranged from intimal xanthoma to pathological intimal thickening, to fibrous cap atheroma, with a mixture of lesions or layering observed. Lumenal occlusion, thrombosis and evidence of recanalization were seen in the carotid artery lesions.

**Fig. 5:**
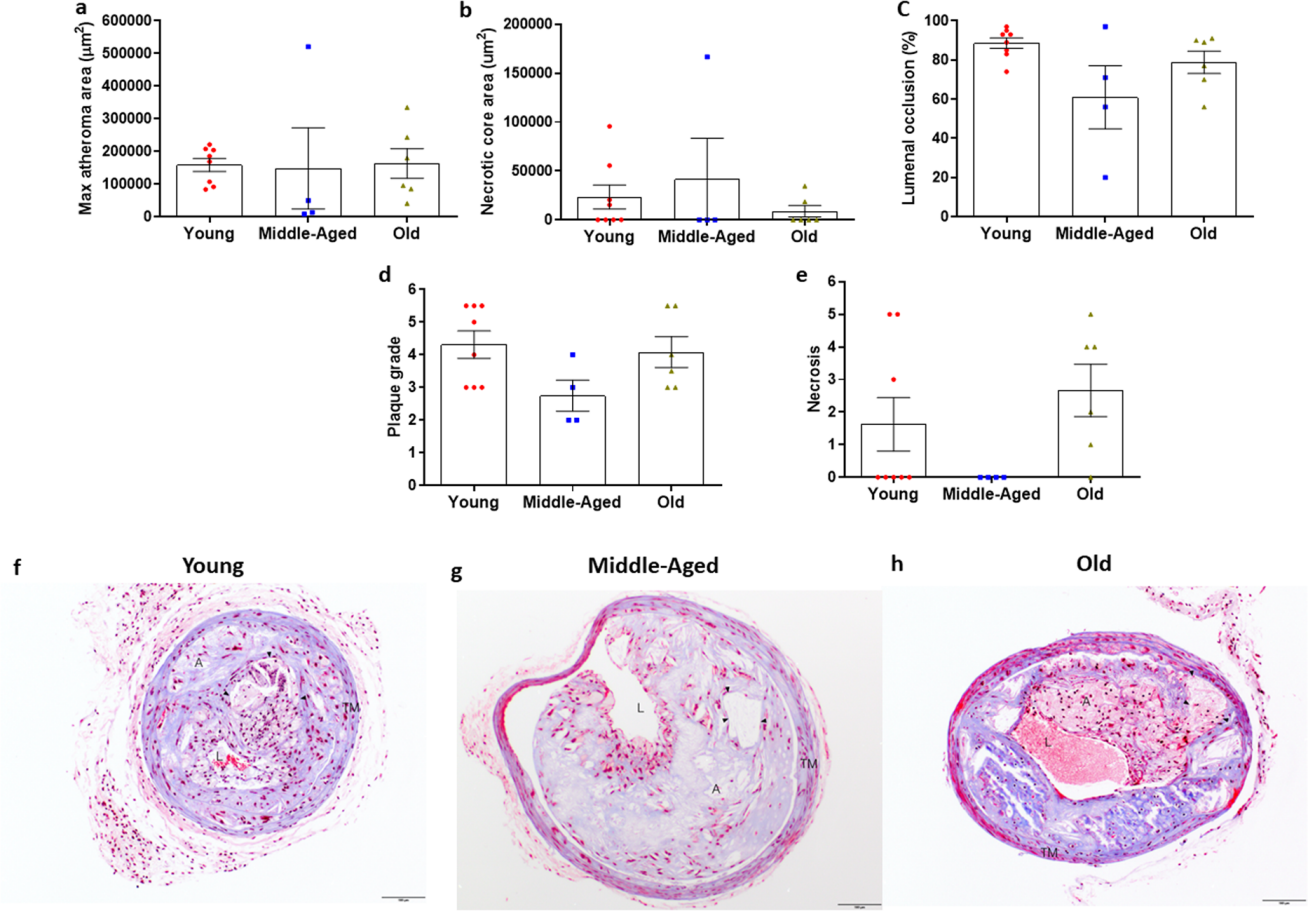
Size and severity of atheromas in the left carotid arteries of young (Y: *N* = 8), middle-aged (MA: *N* = 4) and old (O: *N* = 6) apolipoprotein E knockout (ApoE^−/−^) mice after partial carotid ligation (PCL) and 5 weeks of atherogenic diet (AD). (a) Atheroma area was measured in Masson’s trichrome stained histological sections of the left carotid artery from young, middle-aged and old ApoE^−/−^ mice 5 weeks after PCL and initiation of AD. (b) Area of the necrotic core, when present, in aortic root atheromas from young (*N* = 4), middle-aged (*N* = 1), and old (*N* = 2) mice. (c) Lumenal occlusion expressed as the percent reduction in lumen area, (d) plaque morphology grade and severity score for (e) necrosis in atheromas from the left carotid artery after PCL and 5 wks of AD. Individual data presented are raw values for young, middle-aged, and old mice. Summary data is presented as mean ± SEM. Summary data in panels d and e are median scores. Representative images of Masson Trichrome stained sections of left carotid arteries from young (f), middle-aged (g), and old (h) ApoE^−/−^ mice 5 weeks after PCL and initiation of AD. Lumen (L), atheroma (A), necrotic core (black arrowheads), and tunica media (TM) are indicated. Scale bar indicates 100 μm. To assess differences in plaque grade morphology and severity scores for plaque characteristics, nonparametric or Mann–Whitney Wilcoxon signed rank tests were used

Atheroma area, necrotic core area and plaque severity were assessed in Masson’s trichrome stained paraffin embedded carotid artery sections containing an atherosclerotic plaque. Unlike the spontaneous plaques in the descending aorta, there was no effect of age on size of the atheromas in the left (ligated) carotid artery (Fig. 5a, *P* = 0.984). No atheromas were present in the contralateral right (unligated) carotid artery in any age group. While necrotic cores were present in 25–50% (4 of 8 in young, 1 of 4 in middle-aged, 2 of 6 in old) of atheromas in the arteries after PCL, there was no effect of age on the area of these necrotic cores (Fig. 5b, *P* = 0.542) but a tendency towards differences in percent occlusion of the lumen (Fig. 5c, *P* = 0.06).

Likewise, there was no effect of age on plaque morphology grade in the PCL-induced atheromas (Fig. 5d and Table 1, *P* = 0.125). Although intimal necrosis was not observed in PCL-induced atheromas of middle-aged mice, 37.5% (3 of 8) of young and 83% (5 of 6) of old mouse carotid atheromas demonstrated intimal necrosis (Fig. 5e). However, differences in the severity score for intimal necrosis between young and old mice failed to reach significance (Fig. 5e, Table, *P* = 0.080). There was minimal evidence of, and no differences in, mineralization, intraplaque hemorrhage, thrombosis, recanalization, or inflammation (Table 1). Representative images of carotid artery atheromas are presented in Fig. 5 f–h.

### Circulating lipids in young and old Pcsk9 treated mice

**Fig. 6:**
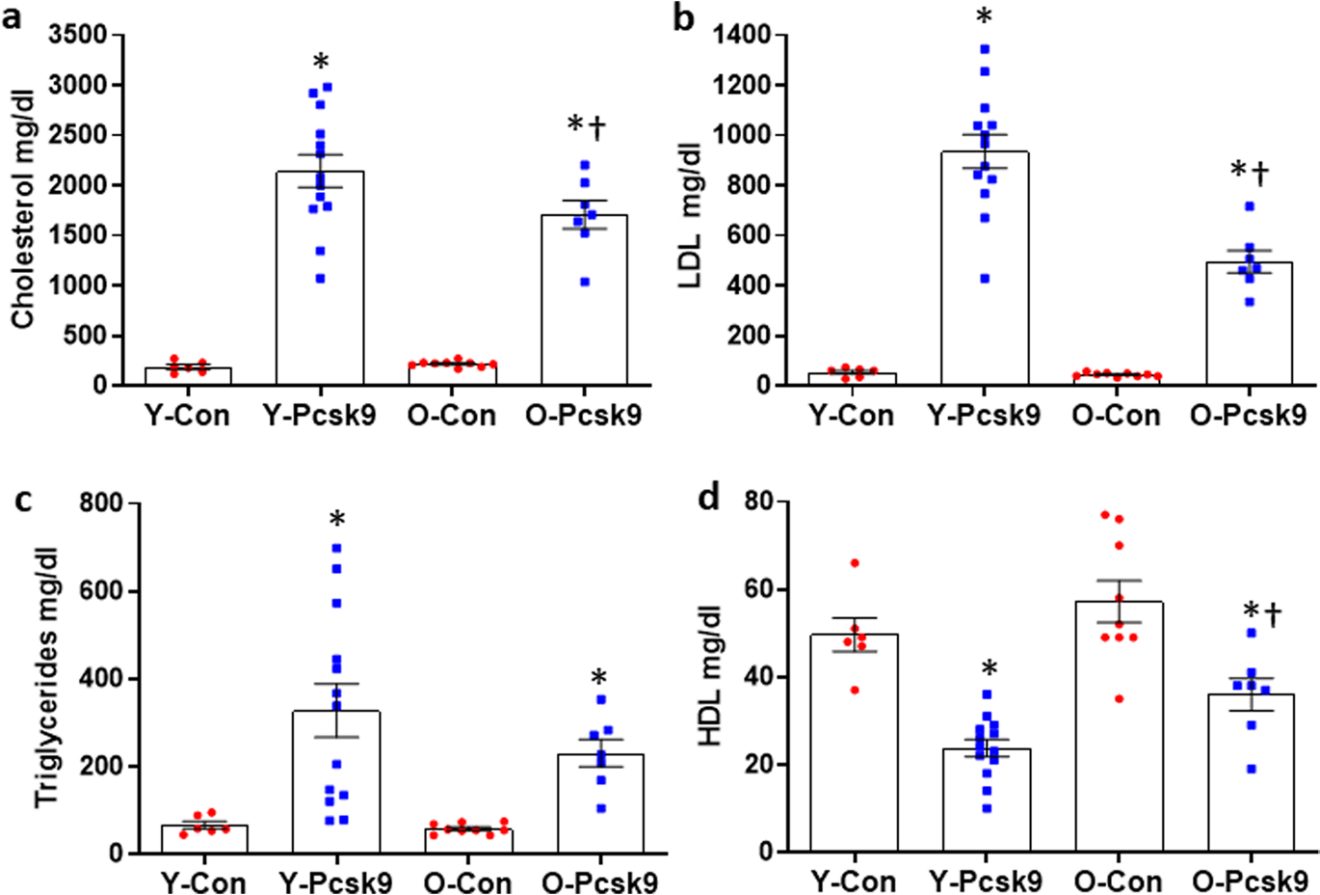
Circulating lipids in young (7 mo, Con *N* = 6, Pcsk9 *N* = 13), or old (19 mo, Con *N* = 9, Pcsk9 = 7) C57BL/6 mice treated with a control or proprotein convertase subtilisin/kexin type 9 (*Pcsk9*) mutant-containing adenoassociated virus (AAV) and fed atherogenic diet for 5 weeks. Total cholesterol (a), LDL cholesterol (b), triglycerides (c), and HDL cholesterol (d) were assessed by Architect ci8200 biochemical analyzer. Individual data presented are raw values for young (Y) and old (O) control (Con)-AAV and Pcsk9-AAV treated mice. Summary data is mean ± SEM, * denotes *p* < 0.05 compared to age-matched Con treated mice, † denotes *p* < 0.05 compared to treatment matched young mice. Data were combined over weeks on AD, a one-way ANOVA with LSD post hoc testing was performed

To assess the atherosclerotic impact of aging on an acute increase in circulating lipids, young and old C57BL/6 mice were treated with a control or Pcsk9 overexpressing adeno-associated virus (AAV). For both young and old mice, total and LDL cholesterol, as well as triglycerides, were higher in Pcsk9 overexpressing compared to age-matched control AAV treated mice (Fig. 6a-c, all *P* ≤ 0.001). However, total (*P* = 0.027) and LDL (*P* < 0.001) cholesterol were lower in old compared to young AAV-Pcsk9 treated mice(Fig. 6a-b). Triglycerides did not differ between young and old AAV-Pcsk9 treated mice (Fig. 6c, *P* = 0.156). HDL cholesterol was lower in Pcsk9 compared to Con treated young and old mice (Fig. 6d, both *P* < 0.001), and HDL cholesterol was higher in old compared with young mice treated with AAV-Pcsk9 (Fig. 6d, *P* = 0.016). Thus, although AAV-Pcsk9-induced hyperlipidemia occurred in both young and old mice, the stimulus for plaque development was modestly blunted in the aged mice.

### Aortic plaques and aortic root atheromas in young and old Pcsk9 treated mice

In descending aortas stained with Sudan IV from Pcsk9 and control treated mice, there was minimal evidence of plaque development in either young or old mice. The mean percent plaque area was less than 0.6% in all groups and there were no differences observed between groups (*P* = 0.275, not shown).

**Fig. 7:**
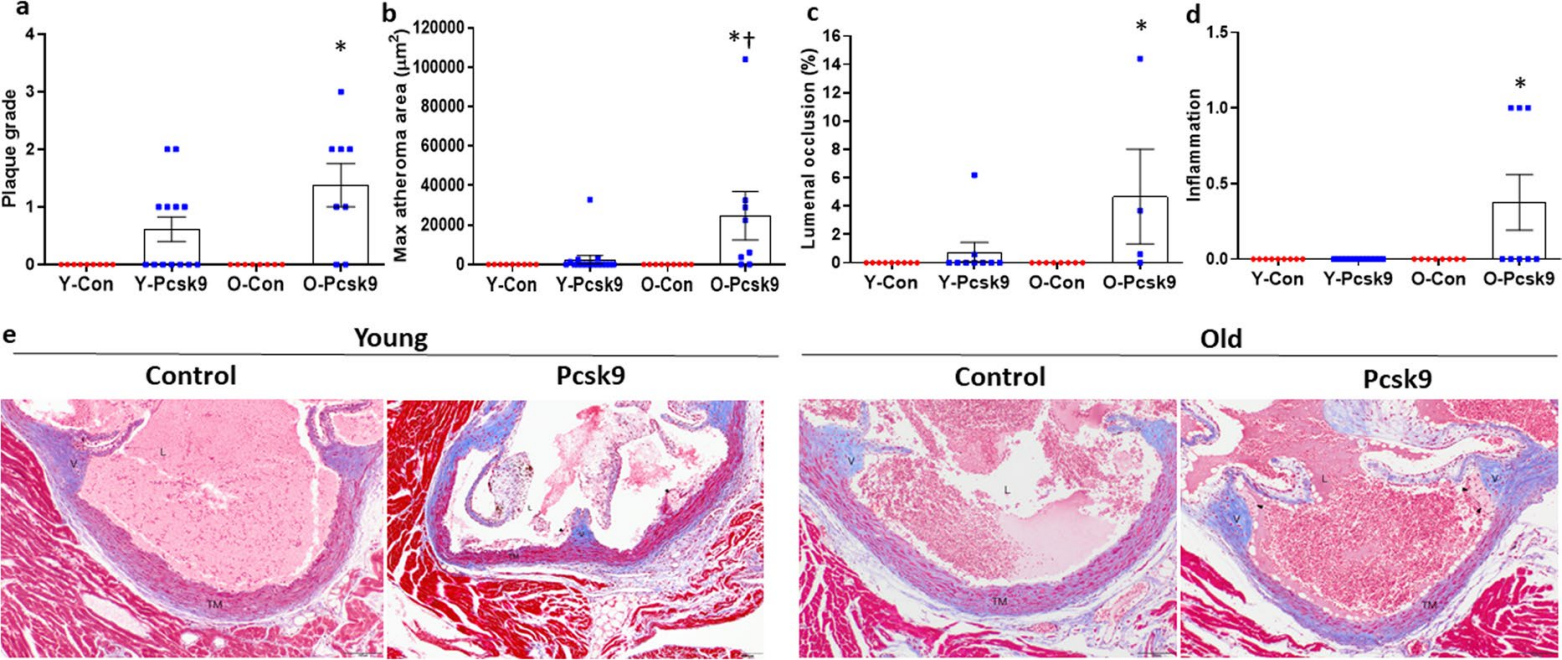
Size and severity of atheromas in the aortic root from young (Y: Con *N* = 9, Pcsk9 *N* = 13–16) and old (O: Con *N* = 8, Pcsk9 *N* = 4–8) C57BL/6 mice treated with either a control (Con) or mutant Pcsk9 containing adeno-associated virus. (a) Plaque grade was measured in Masson’s trichrome stained histological sections of aortic roots from young and old Con and Pcsk9 treated mice fed an atherogenic diet (AD) for 5 weeks after AAV injections (b) Atheroma area was measured from Masson’s trichrome stained slides with multiple aortic root atheroma samples available, maximal area was used in the analyses. (c) Lumenal occlusion, expressed as the percent reduction by atheroma within the aortic root intimal area, measured with Masson’s trichrome-stained slides as well as and (d) severity score for inflammation in aortic root atheromas. Individual data presented are raw values and summary data are mean ± SEM. * denotes *p* < 0.05 compared to age-matched Con treated mice, † denotes *p* < 0.05 compared to treatment matched young mice. (e) Representative images of aortic roots stained with Masson’s trichrome. Lumen (L), valve base (V), tunica media (TM), and intimal lesions (atheromas; black arrowheads) are indicated. Scale bar indicates 100 μm. To assess differences in plaque size and severity scores for plaque characteristics, nonparametric or Mann–Whitney Wilcoxon signed rank tests were used where appropriate

When present, aortic root lesions were primarily intimal xanthomas or pathological intimal thickening. Fibrous cap atheromas were not observed. Compared to control AVV treated mice, plaque morphology grade of aortic root atheromas was higher in old (*P* = 0.005) but not young (*P* = 0.225) Pcsk9 overexpressing mice (Fig. 7a, Table 1). In Masson’s trichrome stained sections of the aortic root, atheromas were present in 31% of young (4 of 13) and 75% of old (6 of 8) Pcsk9 overexpressing mice but were absent in all control treated mice. Aortic root atheroma area was larger (Fig. 7b, *P* = 0.012) resulting in greater lumenal occlusion (Fig. 7c,*P* = 0.025) in the old compared to the young AVV-Pcsk9 treated mice. There were no necrotic cores or fibrous caps present in aortic root atheromas from either the young or the old Pcsk9 mice. Severity score for inflammation differed between groups (Fig. 7d, *P* = 0.008), with a higher severity score in AAV-Pcsk9 compared to control AAV treated old (Table 1, *P* = 0.036), but not young (Table 1, *P* = 1.000) mice; as well as in old compared to young Pcsk9 overexpressing mice (Table 1, *P* = 0.014). However, these atheromas presented with little to no evidence of intimal necrosis, mineralization, intraplaque hemorrhage, or recanalization. Representative images of aortic root atheromas are presented in Fig. 7e. Thus, despite lower circulating cholesterol in old compared to young Pcsk9 AVV treated mice, plaque size and grade at the aortic root were modestly greater in the old mice suggesting an exacerbation of atherosclerotic development in response to hyperlipidemia in the old mice.

### PCL-induced carotid artery lesions in young and old Pcsk9 treated mice

**Fig. 8:**
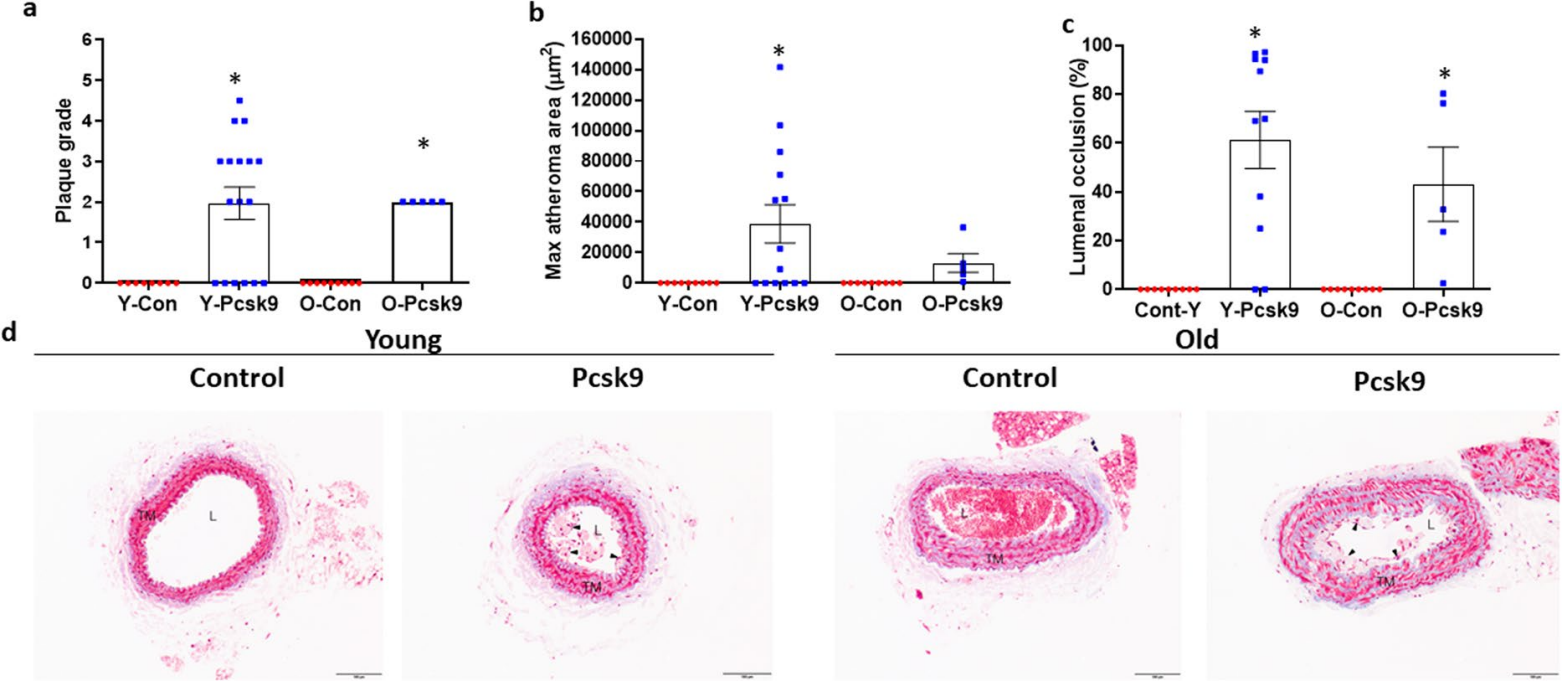
Size and severity of atheromas in the left carotid artery of young (Y: Con *N* = 8–9, Pcsk9 *N* = 11–17) and old (O: Con *N* = 9, Pcsk9 *N* = 5) C57BL/6 mice treated with either a control (Con) or mutant Pcsk9 containing adeno-associated virus after partial carotid ligation (PCL). (a) Plaque morphology grade was scored in Masson’s trichrome stained histological sections of carotid arteries from young and old Con and Pcsk9 overexpressing mice fed 5 weeks after PCL and initiation of atherogenic diet. (b) Atheroma area in the left carotid artery. Maximal area was used in the analysis if multiple sections were available. (c) Lumenal occlusion expressed as a percent of lumen area. Individual data presented are raw values and summary data in panels b and c are mean ± SEM and are median scores in panel a. * denotes *p* < 0.05 compared to age-matched Con mice. (d) Representative images of left carotid arteries stained with Masson’s trichrome (MT). Lumen (L), tunica media (TM), and intimal lesions (atheromas; black arrowheads) are indicated. Scale bar indicates 100 μm. To assess differences in plaque size and severity scores for plaque characteristics, nonparametric or Mann–Whitney Wilcoxon signed rank tests were used where appropriate

Carotid artery lesion morphology ranged from intimal xanthoma, to pathological intimal thickening, to fibrous cap atheroma. Plaque morphology grade was higher in PCL-induced carotid artery lesions of Pcsk9 overexpressing compared to control AAV treated young (*P* = 0.010) and old (*P* = 0.028) mice (Fig. 8a). However, contrary to our hypothesis, there was no difference in plaque morphology grade between young and old Pcsk9 overexpressing mice (Fig. 8a, Table 1, *P* = 1.000). After PCL, there were no atheromas present in the ligated carotid arteries of the control AAV treated mice. Atheromas were present in the carotid arteries of 57% (8 of 14) of the young and 63% (5 of 8) of the old Pcsk9 overexpressing mice. However, neither the size of atheromas (*P* = 0.252) nor percent of lumenal occlusion (*P* = 0.346) differed between young and old Pcsk9 mice (Fig. 8b-c). Necrotic cores were present in 4 of 8 atheromas and fibrous caps were present in 3 of the 8 atheromas observed in young Pcsk9 mice; surprisingly, both were absent in atheromas of old mice.

There was a significant main effect for treatment group in severity score for both intraplaque hemorrhage (*P* = 0.015) and inflammation (*P* = 0.046). In Pcsk9 versus control AAV treated mice, severity score for hemorrhage was higher in old (*P* = 0.021), but not young (*P* = 1.000) mice; whereas severity score for inflammation tended to be higher in young (*P* = 0.074) but not old (*P* = 1.000) mice in the left carotid artery after PCL. There were no differences between young and old Pcsk9 overexpressing mice for either hemorrhage or inflammation severity scores (Table 1, both *P* > 0.231). There were also no differences in any other plaque characteristics (Table 1). Representative images of carotid arteries are presented in Fig. 8d. Despite an age-associated exacerbation in spontaneous lesion development at the aortic root, aging did not impact the atherogenic response to an acute increase in oscillatory shear in the carotid artery.

**Fig. 9:**
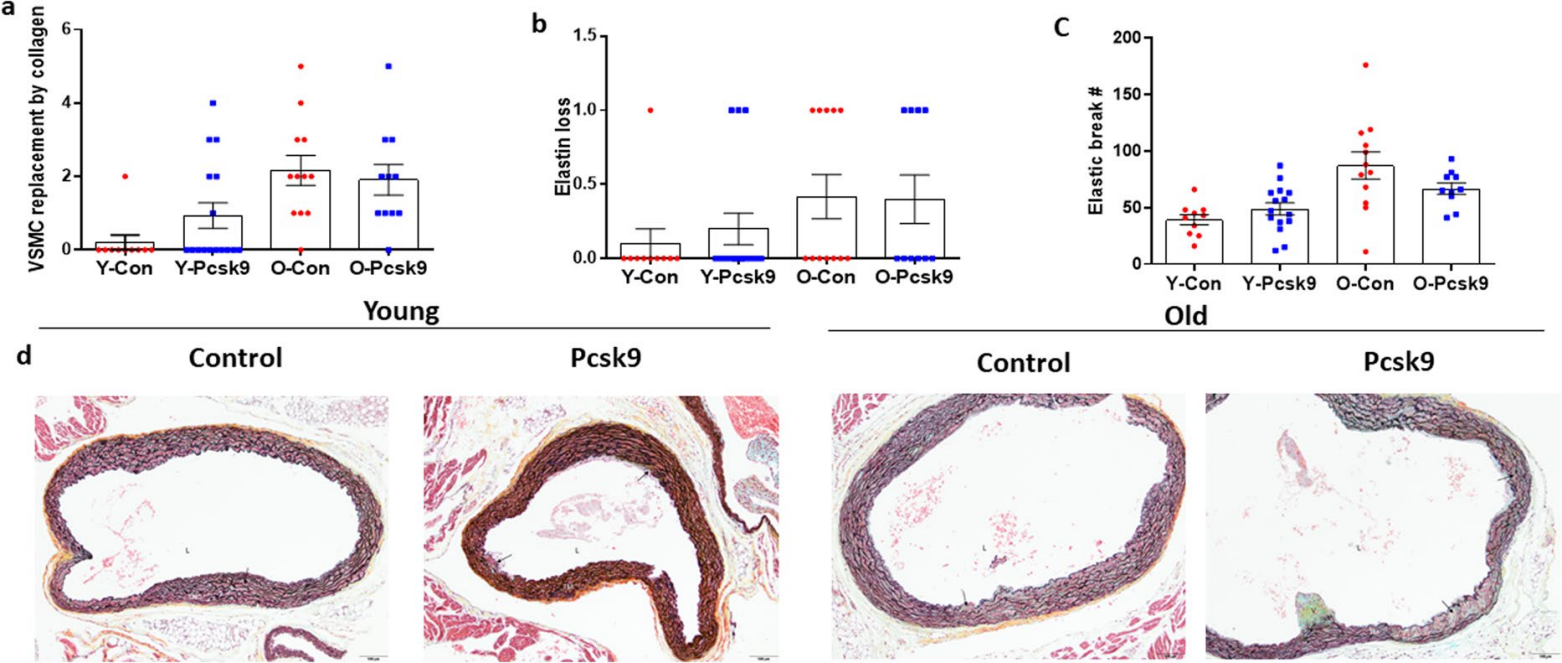
Measures of VSMC replacement by collagen or proteoglycan matrix and elastin loss in the aortic root from young (Y: Con *N* = 10, Pcsk9 *N* = 15–16) and old (O: Con *N* = 12, Pcsk9 *N* = 10–11) C57BL/6 mice treated with either a control (Con) or mutant Pcsk9 containing adeno-associated virus. (a) Area of tunica media VSMC replaced by collagen or proteoglycan matrix was assessed in Movat’s pentachrome-stained histological sections of aortic roots from young and old Con and Pcsk9 treated mice fed an atherogenic diet (AD) for 5 weeks after AAV injections. For samples with multiple aortic root atheroma samples available, maximal area was used in the analyses. (b) Elastin loss score (area of complete loss of elastin), as well as (c) elastin break counts were assessed using Movat’s pentachrome-stained sections. Individual data presented are raw values and summary data is mean ± SEM. (d) Representative images of aortic roots stained with Movat’s pentachrome. Lumen (L), tunica media (TM), valve base (v), VSMC (stained red–orange), proteoglycan (stained aqua blue), collagen (stained yellow) matrix, elastin (laminae stained black), and areas of elastin loss and collagen/proteoglycan deposition (black arrows) are indicated. Scale bar indicates 100 μm. To assess differences in root morphology for plaque characteristics, nonparametric or Mann–Whitney Wilcoxon signed rank tests were used

We further examined morphology of the aortic roots with plaques and found no aging effect on collagen deposition in the media between control and Pcsk9 AAV treated young (Fig. 9a, *P* = 0.138) or old (*P* = 0.634) mice. Similarly, there was no effect of age on elastin loss compared to control treated young (*P* = 0.626) and old (*P* = 0.999) Pcsk9 mice (Fig. 9b). Likewise, there was no change in elastin breaks compared to control treated young (*P* = 0.056) and old (*P* = 0.264) mice (Fig. 9c). Representative Movat’s pentachrome-stained images of aortic root morphology including of VSMCs, collagen, proteoglycan, and elastin in control and Pcsk9 treated young and old mice are presented in Fig. 9d.

## Discussion

In the present study, we sought to determine the impact of aging on spontaneous and oscillatory shear induced atherosclerotic lesions in two atheroprone mouse models, the ApoE^−/−^ mouse and C57BL/6 mouse treated with an AAV to upregulate Pcsk9. To do so, we (a) examined aortic plaque burden by quantifying Sudan IV staining in the longitudinal aorta, (b) assessed the size and morphological grade of atheromas in the aortic root, and (c) assessed the size and morphological grade of atheromas in the carotid artery after induction of oscillatory shear stress by PCL. These measures were performed in young, middle-aged and old ApoE^−/−^ mice and young and old Pcsk9 overexpressing C57BL/6 mice, both fed an atherogenic diet. The novel findings of our study are: (1) Concomitant with higher total and LDL cholesterol, and triglycerides, old age increases aortic plaque burden and the size and severity of aortic root plaques in AD fed ApoE^−/−^ mice independent of number of weeks on diet. However, there was no effect of age on size or severity of oscillatory shear-induced carotid artery atheromas after PCL. (2) Despite higher total and LDL cholesterol in young mice after Pcsk9 treatment, aortic root atheroma size and morphology grade were higher in old compared to young mice, although aging did not impact carotid artery lesion size or grade in Pcsk9 overexpressing mice after PCL.

While rodents are normally resistant to atherosclerotic plaque development, genetically modified strains such as the ApoE^−/−^ mouse demonstrate hyperlipidemia and develop atherosclerotic lesions when fed an AD. Longitudinal studies of the effect of aging in the ApoE^−/−^ model are confounded by length of time mice have been exposed to atherogenic stimuli (hyperlipidemia and low/oscillatory shear stress), making a true evaluation of the impact of aging per se difficult. To begin to overcome this limitation, we sought to use surgical models of induced oscillatory shear. These models can be utilized to induce a more severe plaque morphology in a shorter time than is observed in the aorta of ApoE^−/−^ mice on an atherogenic diet. Such surgical models include both complete and partial ligation of the carotid artery. PCL is achieved by ligating 3 of the 4 caudal branches of the left carotid artery, with the superior thyroid artery left intact. PCL-induced atherosclerotic plaques in ApoE^−/−^ mice demonstrate intraplaque neovessels and cholesterol clefts that are characteristic of advanced human lesions [17]. PCL is preferred to other surgical models of oscillatory shear induced advanced lesion formation, as it allows for a physiologically relevant shear profile without endothelial cell loss. For these reasons, we utilized PCL in the atheroprone ApoE knockout model as a surgical tool to evaluate the impact of aging on the atherogenic response to a physiologically relevant oscillatory shear stimulus.

Furthermore, while the ApoE^−/−^ model is well established, exploring mechanisms of plaque development using other novel genetic models requires time-consuming and costly crossbreeding of strains. Treatment with an AAV to upregulate Pcsk9 in the liver is a recently developed atherogenic model that can induce hyperlipidemia and lesion formation in mice fed an AD without the requirement of crossbreeding into an atheroprone background [18, 19]. The Pcsk9 model of atherosclerosis allows for the study of atherosclerotic plaque development in otherwise athero-protected mouse models [18, 20]. Pcsk9 is upregulated in patients with hypertension and hypercholesterolemia and is linearly associated with a larger fraction of an atheroma containing a necrotic core fraction in patients, but is not associated with the presence of a thin cap fibrous atheroma, overall plaque burden or plaque volume [21]. Transgenic ApoE^−/−^ mice also overexpressing human Pcsk9, demonstrate larger atherosclerotic lesions with greater monocyte infiltration compared to Pcsk9 wildtype ApoE^−/−^ mice despite no change in circulating lipids [22]. In control mouse strains, hyperlipidemia is rapidly induced by AAV-mediated increases in hepatic Pcsk9 expression and persists for at least one year [18]. Atherosclerotic plaques will develop spontaneously in atheroprone regions of the aorta of AD fed mice after Pcsk9 overexpression by an AAV [18, 20]. Indeed, aortic arch plaques are present [20] in young Pcsk9 mice fed an AD 3 months post Pcsk9 treatment and this plaque development can be accelerated when combined with PCL surgery, with plaques present in the ligated artery within 3 weeks [20].

Thus, this model is an attractive alternative that requires further elucidation, as the size and severity of lesions obtained using this method have been demonstrated to be less severe than the ApoE^−/−^ strain and, at present, it is unclear if this method will allow for evaluation of lipogenic plaque development in the descending aorta. Here, we aimed to utilize this gene therapy approach to better explore the impact of aging on the atherogenic response to an acute hyperlipidemic stimulus in the absence (spontaneous aortic root atheroma) or presence of induced oscillatory shear (i.e., carotid artery after PCL). Thus, in the present study, we employed varied approaches in an attempt to better address our overarching question: What is the impact of aging on atherosclerotic disease development? First, we used multiple lengths of exposure to AD (3, 5, and 8 weeks) across the lifespan of the ApoE^−/−^ mouse. Second, we utilized PCL to induce an acute atherogenic oscillatory shear stimulus in ApoE^−/−^ mice across the lifespan. Third, in combination with PCL, we utilized AAV-mediated upregulation of Pcsk9 and AD to acutely induce hyperlipidemia in young and old mice. Together, these methods allowed us to better isolate aging and evaluate its impact on both spontaneous and oscillatory shear induced lesions.

We first evaluated the impact of age and diet duration on spontaneous plaque development in the longitudinal aorta and in the aortic roots of ApoE^−/−^ mice. We found that for all three diet durations, aortic plaque burden and aortic root atheroma area were higher in old mice compared to young mice. We further found that, with the exception of aortic root atheroma area in young mice, spontaneous plaque burden and aortic root atheroma area were higher after 8 compared to 3 weeks AD in all three age groups. The effect of aging on susceptibility to AD was associated with increased circulating cholesterol in old mice compared to both young and middle-aged mice. While this finding is consistent with a previous study that demonstrated increased circulating cholesterol and lesion development in normal chow fed ApoE^−/−^ mice [16], it remains unclear if aged ApoE^−/−^ mice in the present study demonstrated an exacerbated proatherogenic response or if increased plaque size and severity is a consequence of either higher circulating cholesterol with advancing age or the prolonged exposure to hyperlipidemia that would have existed even before initiation of AD in the middle-aged and older mice. Nevertheless, these data suggest an exaggerated atherogenic response to hyperlipidemia with advancing age that required further elucidation.

To further address this question, we utilized PCL to induce a pro-atherogenic stimulus to an artery that does not develop atherosclerotic lesions under normal flow conditions. When used in an atheroprone mouse model such as the ApoE^−/−^ mouse, this procedure induces rapid atherosclerotic lesion development [17] and reductions in lumen size in proximal regions of the artery close to the site of ligation, with smaller lesions present in regions of the carotid artery further upstream from the ligation [23]. Here, we found that PCL was effective at inducing lesions in the otherwise largely protected carotid artery in the mouse, but there was no effect of aging to exacerbate the size and severity of atheromas. The results from this acute pro-atherogenic oscillatory shear stimulus suggest that either aging per se does not exacerbate atherogenesis, that there is a dampened pro-atherogenic response to oscillatory shear stress with aging that modulates atherogenesis, or that our methods have reached a ceiling effect as occlusion was nearly complete in all age groups. This is in agreement with previous studies [15, 24], in which it was found that both partial and full ligation of the carotid artery induced similar remodeling between young and old mice. Although determining if there is a differential pro-atherogenic response to oscillatory shear with aging requires further study, previous work from our laboratory has demonstrated that there is an age-associated reduction in proliferation in aged vascular smooth muscle cells, such that in response to either fatty acid treatment (to model hyperlipidemia) or in response to treatment with conditioned media from fatty acid treated endothelial cells, there is reduced proliferation in cells from old compared to young mice [15]. Such a reduction in proliferative potential with aging may limit plaque growth in the setting of hyperlipidemia. Likewise, in a study utilizing carotid ligation, there was less remodeling in response to decreased blood flow and shear stress in adult (~ 200 g) compared to juvenile (~ 100 g) rats [25]. The impact of aging on the response of endothelial cells to oscillatory shear and how this may interact with vascular smooth muscle and immune cells in the setting of atherosclerosis requires further elucidation.

Using Pcsk9 treatment in the absence of PCL, a recent report demonstrated increased lesion size in the brachiocephalic artery, but not aortic root, of 18–19 month compared to 2–3 month old C57Bl/6 mice treated with AAV-Pcsk9 and fed an AD for 10 weeks [26]. In the present study, we found a larger aortic root atheroma with a more severe plaque morphology grade 5 weeks after Pcsk9 treatment and initiation of AD in old compared to young C57BL/6 mice. In agreement with Tyrrell et al. [26], we also found that total cholesterol was higher in the young compared to the old Pcsk9 overexpressing mice. Together, these findings support an age-associated exacerbation of atheroma development in response to a hyperlipidemic stimulus in the aortic root, as lower circulating cholesterol is associated with larger, more severe aortic root atheromas in aged mice.

Interestingly, similar to what was found in the ApoE^−/−^ mice, the effect of aging to exacerbate size and severity of atherosclerotic lesions in the Pcsk9 model is lost in the carotid arteries after PCL. While oscillatory shear induced lesions in the carotid artery after PCL appeared to be both larger and more severe than what was observed in the aortic roots of the same animal in the young ApoE^−/−^ and Pcsk9 mice, plaque size and severity did not appear to differ between these sites in the middle-aged and old mice, suggesting that aging differentially impacts atherogenesis depending on the predominant underlying pro-atherogenic stimulus. While the reasons for reduced atherogenesis in response to oscillatory shear in old mice are unclear, oscillatory shear stress has been demonstrated to be elevated in conduit arteries with advancing age [27–29] and, as a result, downregulation of shear sensing pathways may attenuate the pro-atherogenic responses observed. While this is an unsupported notion, similar results with aging in both of the models utilized in the present study strengthens the evidence for an age-associated reduction in pro-atherogenic response to oscillatory shear. While this requires further study, the similar results with aging in both models utilized in the current study supports an age-associated reduction in the pro-atherogenic response to oscillatory shear.

### Summary

Although advancing age appears to increase atherogenesis in response to hyperlipidemia, there does not appear to be an exacerbation of the pro-atherogenic response to oscillatory shear. The mechanisms for this disparate atherogenic response with aging are unclear but may result from a reduced ability of the endothelium to sense and/or respond to changes in shear stress with advancing age, a possibility requiring further elucidation. Nevertheless, in response to hyperlipidemia, aged arteries develop larger and more advanced lesions in anatomical areas of disturbed flow such as the aortic root, supporting an age-associated exacerbation of atherogenesis. Future studies should aim to elucidate the mechanisms of this exaggerated response.

## Methods

All animal procedures conform to the Guide for the Care and Use of Laboratory Animals [30] and were approved by the Institution Animal Care and Use Committees at the University of Utah and Veteran’s Affairs Medical Center-Salt Lake City (VAMC-SLC).

We utilized two models of atherosclerosis, ApoE^−/−^ mice from Jackson Laboratory Inc. (B6.129P2-*Apoetm1Unc*/J) [16, 31] and C57BL/6 mice treated with an AAV containing mutant Pcsk9. This treatment upregulates Pcsk9 in the liver and induces sustained hyperlipidemia after a single injection [18, 20].

### ApoE^−/−^ model

**Fig. 10:**
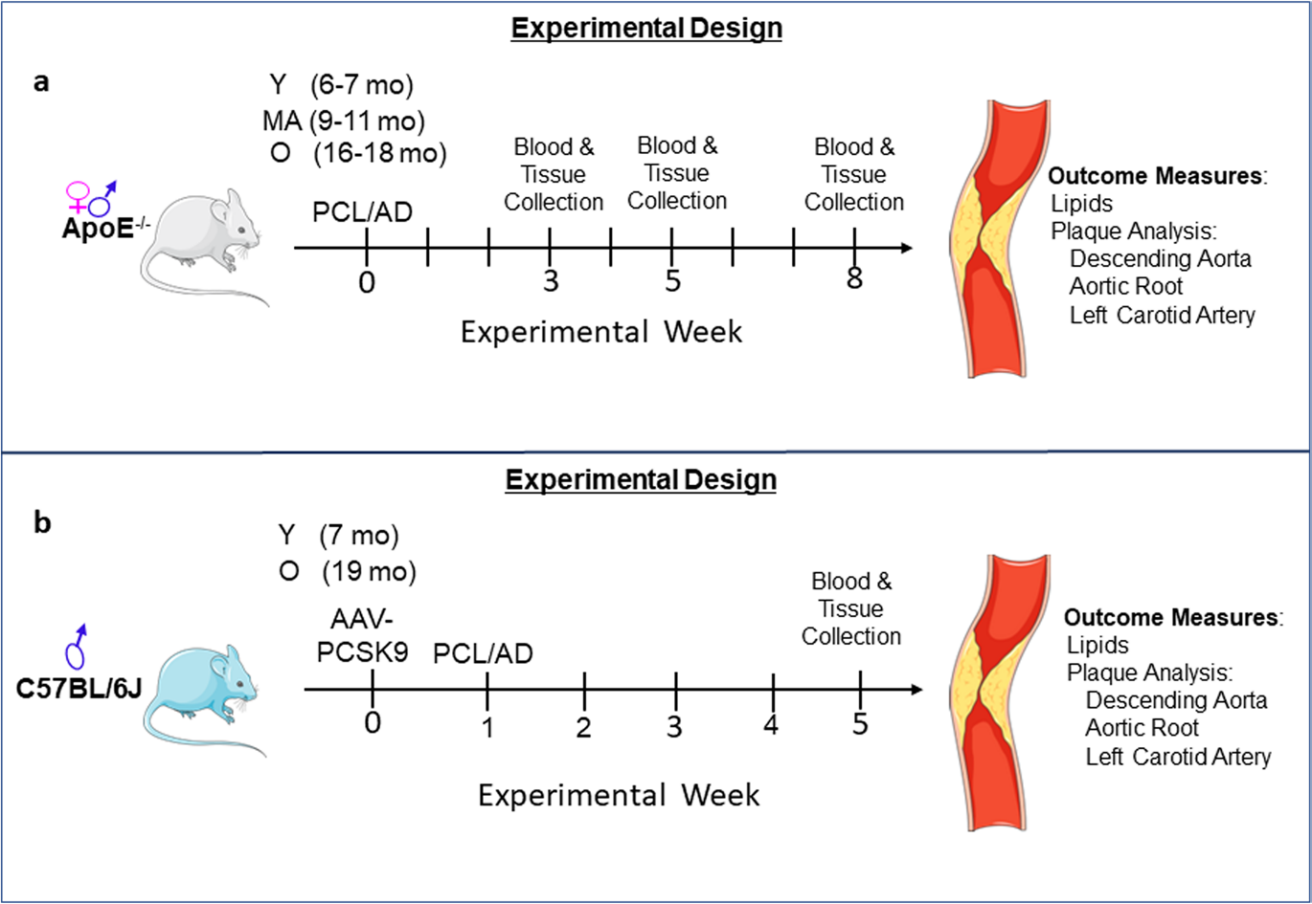
Schematic representation of study design. (a) Experimental design in ApoE^−/−^ mouse model (b) Experimental design for C57BL/6 J mouse model. Y: young, MA: middle age, O: old; PCL: partial carotid ligation, AD: atherogenic diet, 3, 5 and 8 wks: weeks, AAV-Pcsk9: adeno-associated virus—proprotein convertase subtilisin/kexin type 9

Experimental mice and a breeding pair of ApoE^−/−^ mice were obtained from Jackson Laboratory Inc. (B6.129P2-*Apoetm1Unc*/J). A colony was maintained in the VAMC-SLC vivarium to supply additional experimental mice. Young (6–7 mo), middle-aged (9–11 mo) and old (16–18 mo) male and female ApoE^−/−^ mice were fed an atherogenic diet (AD: 37.1% Fat, Envigo Teklad Custom Diet, Cocoa Butter Diet and Purina Mouse Chow, cat # TD.88051) for 3, 5 or 8 weeks prior to euthanasia. PCL was performed on a subset of young, middle-aged and old mice to induce oscillatory shear stress in the left carotid artery. The surgery was performed as previously described [17], AD was initiated at the time of surgery and continued for 5 weeks until euthanasia for tissue collection (Fig. 10a).

### AAV-Pcsk9 model

Young (7 mo) and old (19 mo) male C57BL/6 mice were obtained from Charles River Inc. and from the National Institute on Aging, aged rodent colonies maintained at Charles River. A hyperlipidemic phenotype was induced in C57BL/6 mice by injection of a single dose (1X10^11^ viral genome copies, retro-orbital iv injection, in 100 µl PBS) of an AAV containing an overexpressing Pcsk9 mutant (Vector Biolabs, Catalog # AAV-268246) or a scrambled vector (Vector Biolabs, Catalog # VB2008) (Con, 1X10^11^ viral genome copies, retro-orbital iv injection, in 100 µl PBS) [20]. One week after injection with the Pcsk9 or Con AAV, PCL was performed to induce oscillatory shear stress in the left carotid artery as previously described [17]. AD was initiated at the time of surgery and continued for 5 weeks until euthanasia for tissue collection (Fig. 10b).

### Tissue collection, preparation and analysis

Euthanasia was performed by exsanguination via cardiac puncture while mice were maintained under isoflurane anesthesia. Collected blood was centrifuged (2500 RPM, 4^0^ C, 15 min), and serum collected in a clean microcentrifuge tube and stored at -80^0^C until testing. Serum lipid profile was assessed by Architect ci8200 (Abbott, USA) biochemical analyzer. Total cholesterol, total triglycerides, LDL and HDL levels were measured by using dedicated on-board reagents from Abbott Laboratories (Cat#7D62-21, 7D74-21, 1E31-20 and 3k33-22). To assess the size and severity of the lipogenic plaques in the descending aorta, aortas with heart and carotid arteries attached were excised, formalin fixed, cleared of surrounding tissues, and digital images captured. The heart and aortic arch with attached carotid arteries were removed and saved for processing, staining and analyses of the aortic root. The remainder of the aorta was Sudan IV stained to visualize fatty lesions. Digital images of the whole aorta were collected with the aid of a dissecting microscope and percent plaque area was assessed using ImageJ.

Deidentified fixed heart/aortic arch/carotid artery samples were evaluated by an ACVP-board certified veterinary pathologist. The pathologist identified and excised areas of the aortic roots and carotid arteries that contained atheromas and oversaw the paraffin embedding and sectioning of these lesions. Sections were stained with Masson’s trichrome (MT) and/or Movat’s pentachrome stains (performed by HistoTox Labs) and imaged for evaluation of atheroma area and morphologic pathology according to modified American Heart Association classifications [32, 33]. Plaque morphology grades were assigned based on the lesion category: 1 = intimal thickening, 2 = intimal xanthoma, 3 = pathological intimal thickening, 3.5 = intimal thickening with erosion, 4 = fibrous cap atheroma, 4.5 = fibrous cap atheroma with erosion, 5 = thin fibrous cap atheroma, 5.5 = plaque rupture, 6 = calcified nodule, 7 = fibrocalcific plaque [32]. Aortic roots and lesions from left carotid arteries were also assessed for minimum and maximum fibrous cap thickness, and, necrotic core area, when appropriate (i.e., fibrous cap measures only for plaque grades 4 and 5). In addition, atheromas were assigned a severity score (0 = absent, 1 = minimal, 2 = mild, 3 = moderate, 4 = marked, 5 = severe) for relevant plaque characteristics including intimal necrosis, mineralization, intraplaque hemorrhage, recanalization, and inflammation. The aortic sections used for assessment of collagen and elastin after Movat’s pentachrome staining, were distal to those sampled for plaque analyses in order to ensure the presence of an intact tunica media.

### Statistical analyses

To assess differences between age groups and diet durations for measures of lumenal occlusion, plaque burden and plaque size, a two-way ANOVA was performed and least square differences (LSD) post hoc testing when appropriate. When these data were combined over weeks on AD, a one-way ANOVA with LSD post hoc testing were performed. Data are presented as both raw values and means ± SEM. To assess differences in plaque grade morphology and severity scores for plaque characteristics, nonparametric or Mann–Whitney Wilcoxon signed rank tests were used where appropriate. Data are presented as raw values and median scores.

## Supplementary Information

Below is the link to the electronic supplementary

Figure S1. Analysis of different diet durations in each age group in the circulating lipids of young (6.6 ± 0.1 mo, *N* = 20), middle-aged (10.1 ± 0.2 mo, *N* = 25) or Old (16.9 ± 0.1 mo, *N* = 11) apolipoprotein E knockout (ApoE^−/−^) mice fed an atherogenic diet (AD) for 3, 5, or 8 weeks. Total cholesterol (a), LDL cholesterol (b), triglycerides (c), and HDL cholesterol (d) were assessed by an Architect ci8200 biochemical analyzer. Individual data presented are raw values for young, middle-aged, and old mice after 3 (black circles), 5 (blue squares), and 8 (red triangles) wk AD. Individual data presented are raw values and summary data is mean ± SEM. * denotes *p* < 0.05 compared to young when data is combined over weeks on diet, ‡ denotes difference from 3 wks within age group. To assess difference between age groups and diet duration for measures, a two-way ANOVA was performed and LSD post hoc testing. (PNG 57 kb)

High resolution image (TIF 109 kb)

Figure S2. Measures of sub-analysis of sex difference in the atherogenesis in the aortic root of young (6.6 ± 0.1 mo), middle-aged (10.1 ± 0.2 mo) or Old (16.9 ± 0.1 mo) apolipoprotein E knockout (ApoE^−/−^) mice fed an atherogenic diet (AD) for 3, 5, or 8 weeks. (a) Atheroma area was measured in Masson’s trichrome stained histological sections of aortic roots from young (*N* = M/F:22/9), middle-aged (*N* = M/F:9/3) and old (*N* = M/F:14/15) ApoE^−/−^ mice after 3, 5 or 8 weeks on AD (b) Aortic root atheroma area in young (*N* = M/F:15/7), middle-aged (*N* = M/F:9/3), and old (*N* = M/F:14/15) mice when data is combined over weeks on AD and (c) Plaque morphology grade and severity score in young (M/F:15/7), middle-aged (M/F:9/3), and old (M/F:14/14) mice when data is combined over weeks on AD. Individual data presented are raw values and summary data is mean ± SEM for panel a and b and median score for panel c. * denotes *p* < 0.05 difference within age group of males when data is combined over weeks on diet, † denotes *p* < 0.05 sex difference with age when data is combined over weeks on diet. To assess differences in root morphology for plaque characteristics, nonparametric or Mann–Whitney Wilcoxon signed rank tests were used. (PNG 49 kb)

High resolution image (TIF 97 kb)

## Data Availability

The data that support this paper and other findings of this study are available from the corresponding author upon reasonable request.

## Abbreviations

AD: Atherogenic diet
PCL: Partial carotid ligation
AAV: Adeno-associated virus
*Pcsk9*: Proprotein convertase subtilisin/kexin type 9
ApoE^−^^/^^−^: Apolipoprotein E knockout
