# Supplementary figures and images for: Advancing age increases the size and severity of spontaneous atheromas in mouse models of atherosclerosis
Source: GeroScience. 2023 Apr 22;45(3):1913–31. doi: 10.1007/s11357-023-00776-8 (PMC10400524; doi:10.1007/s11357-023-00776-8)

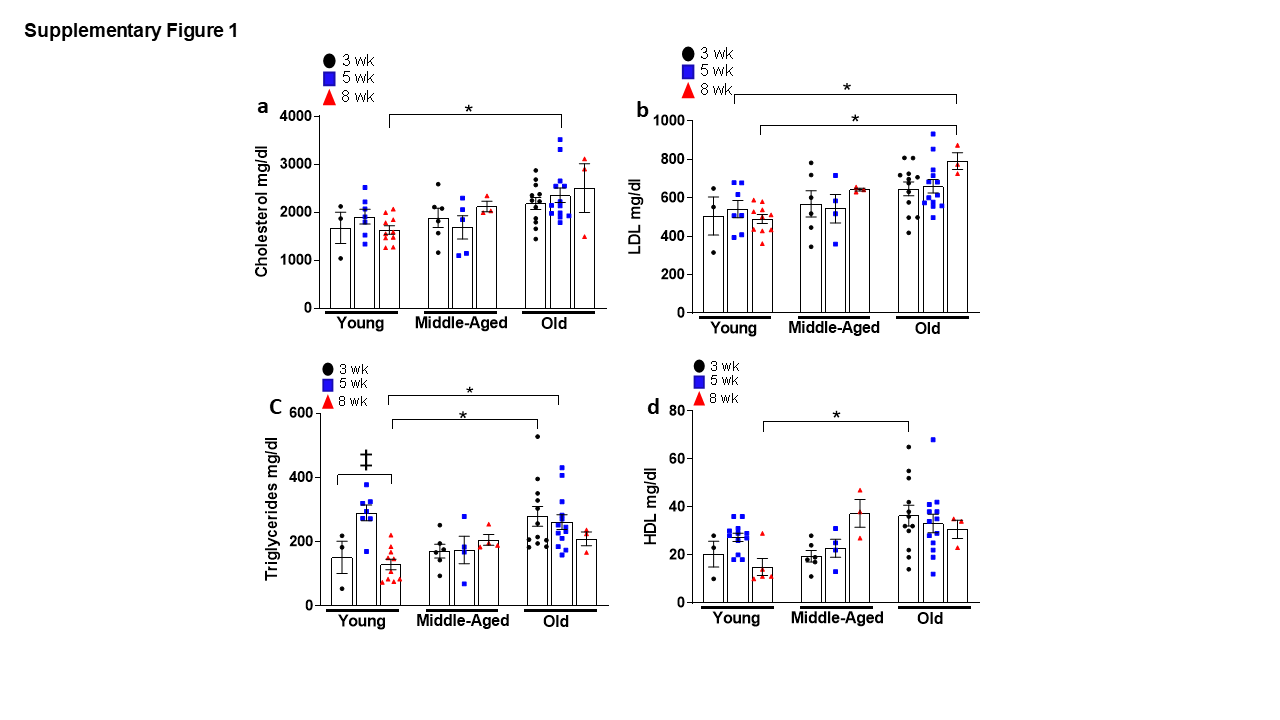

Supplement: Supplementary file 1 — Figure S1. Analysis of different diet durations in each age group in the circulating lipids of young (6.6 ± 0.1 mo, N = 20), middle-aged (10.1 ± 0.2 mo, N = 25) or Old (16.9 ± 0.1 mo, N = 11) apolipoprotein E knockout (ApoE−/−) mice fed an atherogenic diet (AD) for 3, 5, or 8 weeks. Total cholesterol (a), LDL cholesterol (b), triglycerides (c), and HDL cholesterol (d) were assessed by an Architect ci8200 biochemical analyzer. Individual data presented are raw values for young, middle-aged, and old mice after 3 (black circles), 5 (blue squares), and 8 (red triangles) wk AD. Individual data presented are raw values and summary data is mean ± SEM. * denotes p < 0.05 compared to young when data is combined over weeks on diet, ‡ denotes difference from 3 wks within age group. To assess difference between age groups and diet duration for measures, a two-way ANOVA was performed and LSD post hoc testing. (PNG 57 kb) [file 11357_2023_776_Fig11_ESM.png]

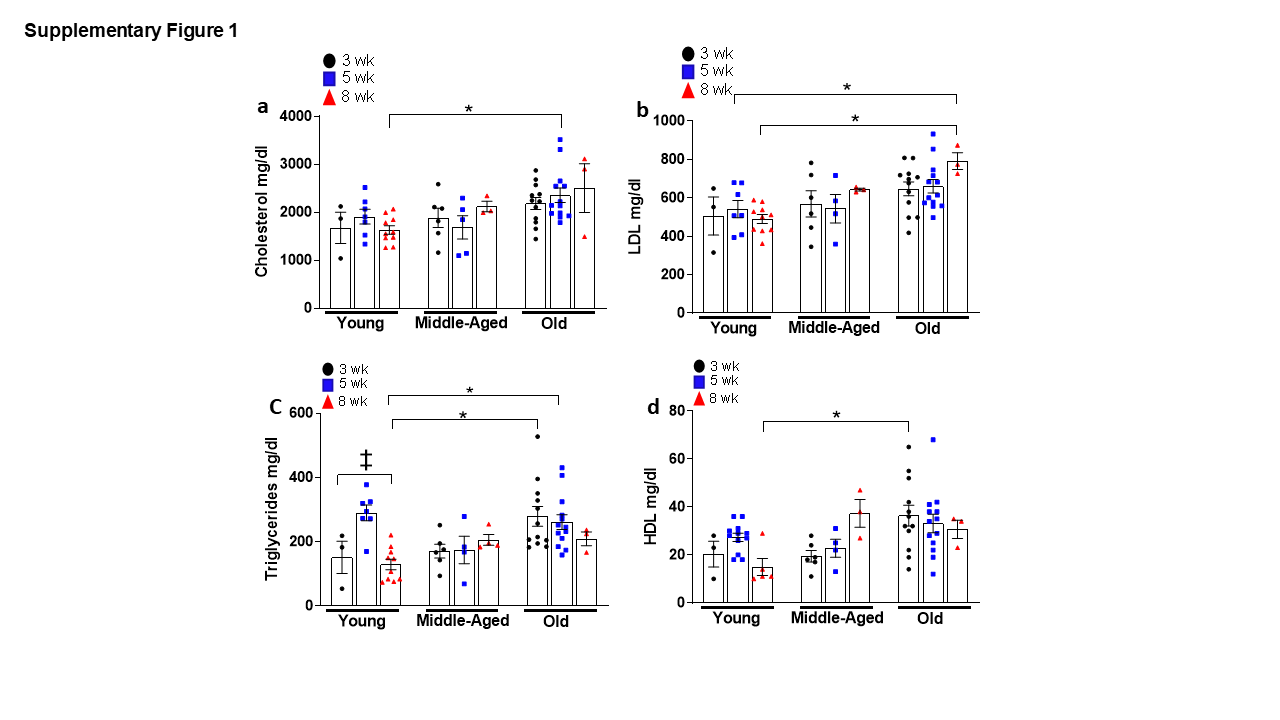

Supplement: Supplementary file 2 — High resolution image (TIF 109 kb) [file 11357_2023_776_MOESM1_ESM.tif]

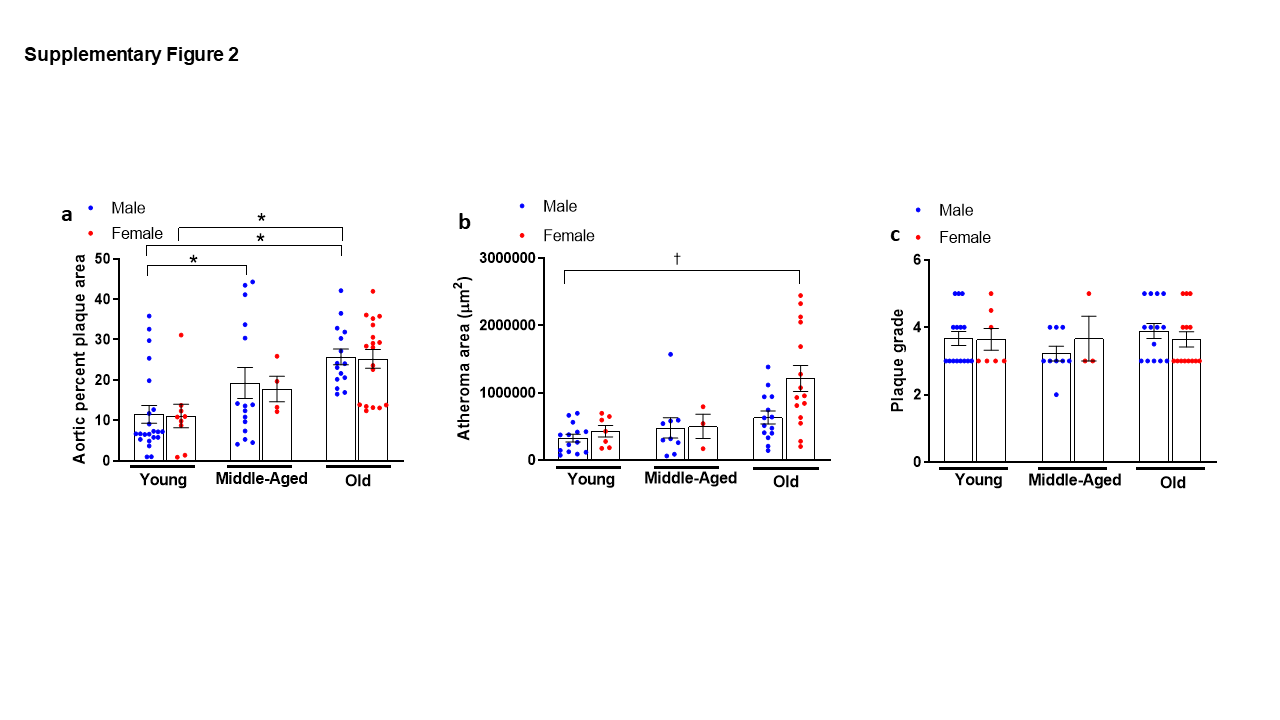

Supplement: Supplementary file 3 — Figure S2. Measures of sub-analysis of sex difference in the atherogenesis in the aortic root of young (6.6 ± 0.1 mo), middle-aged (10.1 ± 0.2 mo) or Old (16.9 ± 0.1 mo) apolipoprotein E knockout (ApoE−/−) mice fed an atherogenic diet (AD) for 3, 5, or 8 weeks. (a) Atheroma area was measured in Masson’s trichrome stained histological sections of aortic roots from young (N = M/F:22/9), middle-aged (N = M/F:9/3) and old (N = M/F:14/15) ApoE−/− mice after 3, 5 or 8 weeks on AD (b) Aortic root atheroma area in young (N = M/F:15/7), middle-aged (N = M/F:9/3), and old (N = M/F:14/15) mice when data is combined over weeks on AD and (c) Plaque morphology grade and severity score in young (M/F:15/7), middle-aged (M/F:9/3), and old (M/F:14/14) mice when data is combined over weeks on AD. Individual data presented are raw values and summary data is mean ± SEM for panel a and b and median score for panel c. * denotes p < 0.05 difference within age group of males when data is combined over weeks on diet, † denotes p < 0.05 sex difference with age when data is combined over weeks on diet. To assess differences in root morphology for plaque characteristics, nonparametric or Mann–Whitney Wilcoxon signed rank tests were used. (PNG 49 kb) [file 11357_2023_776_Fig12_ESM.png]

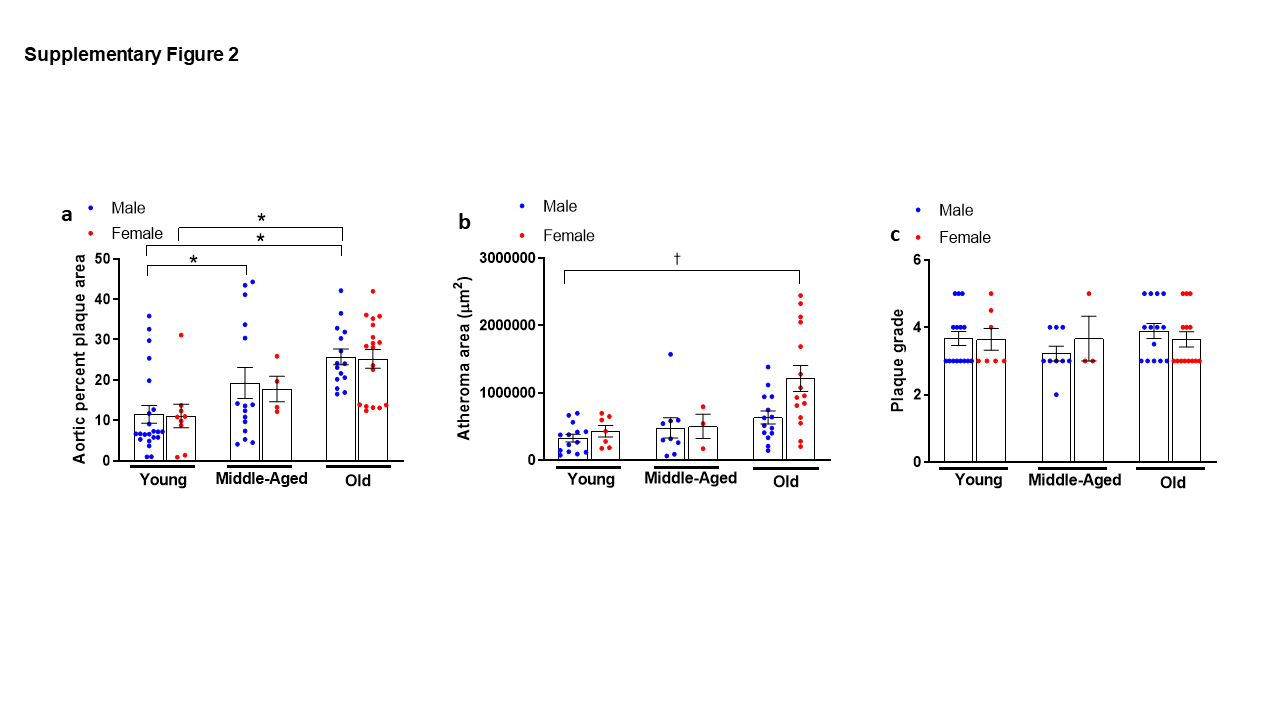

Supplement: Supplementary file 4 — High resolution image (TIF 97 kb) [file 11357_2023_776_MOESM2_ESM.tif]
